# Salt stress improves thermotolerance and high-temperature bioethanol production of multi-stress-tolerant *Pichia kudriavzevii* by stimulating intracellular metabolism and inhibiting oxidative damage

**DOI:** 10.1186/s13068-021-02071-0

**Published:** 2021-11-25

**Authors:** Chunsheng Li, Qiuying Liu, Yueqi Wang, Xianqing Yang, Shengjun Chen, Yongqiang Zhao, Yanyan Wu, Laihao Li

**Affiliations:** 1 Key Laboratory of Aquatic Product Processing, Ministry of Agriculture and Rural Affairs, National R&D Center for Aquatic Product Processing, South China Sea Fisheries Research Institute, Chinese Academy of Fishery Sciences, Guangzhou, 510300 China; 2 Co-Innovation Center of Jiangsu Marine Bio-Industry Technology, Jiangsu Ocean University, Lianyungang, 222005 China; 3 Collaborative Innovation Center of Seafood Deep Processing, Dalian Polytechnic University, Dalian, 116034 China

**Keywords:** *Pichia kudriavzevii*, Thermotolerance, Bioethanol production, Cross-protection, Salt stress, Metabolic network, Oxidative stress

## Abstract

**Background:**

High-temperature bioethanol production benefits from yeast thermotolerance. Salt stress could induce obvious cross-protection against heat stress of *Pichia kudriavzevii*, contributing to the improvement of its thermotolerance and bioethanol fermentation. However, the underlying mechanisms of the cross-protection remain poorly understood.

**Results:**

Salt stress showed obvious cross-protection for thermotolerance and high-temperature ethanol production of *P. kudriavzevii* observed by biomass, cell morphology and bioethanol production capacity. The biomass and ethanol production of *P. kudriavzevii* at 45 °C were, respectively, improved by 2.6 and 3.9 times by 300 mmol/L NaCl. Metabolic network map showed that salt stress obviously improved the key enzymes and intermediates in carbohydrate metabolism, contributing to the synthesis of bioethanol, ATP, amino acids, nucleotides, and unsaturated fatty acids, as well as subsequent intracellular metabolisms. The increasing trehalose, glycerol, HSPs, and ergosterol helped maintain the normal function of cell components. Heat stress induced serious oxidative stress that the ROS-positive cell rate and dead cell rate, respectively, rose from 0.5% and 2.4% to 28.2% and 69.2%, with the incubation temperature increasing from 30 to 45 °C. The heat-induced ROS outburst, oxidative damage, and cell death were obviously inhibited by salt stress, especially the dead cell rate which fell to only 20.3% at 300 mmol/L NaCl. The inhibiting oxidative damage mainly resulted from the abundant synthesis of GSH and GST, which, respectively, increased by 4.8 and 76.1 times after addition of 300 mmol/L NaCl. The improved bioethanol production was not only due to the improved thermotolerance, but resulted from the up-regulated alcohol dehydrogenases and down-regulated aldehyde dehydrogenases by salt stress.

**Conclusion:**

The results provide a first insight into the mechanisms of the improved thermotolerance and high-temperature bioethanol production of *P. kudriavzevii* by salt stress, and provide important information to construct genetic engineering yeasts for high-temperature bioethanol production.

**Graphical Abstract:**

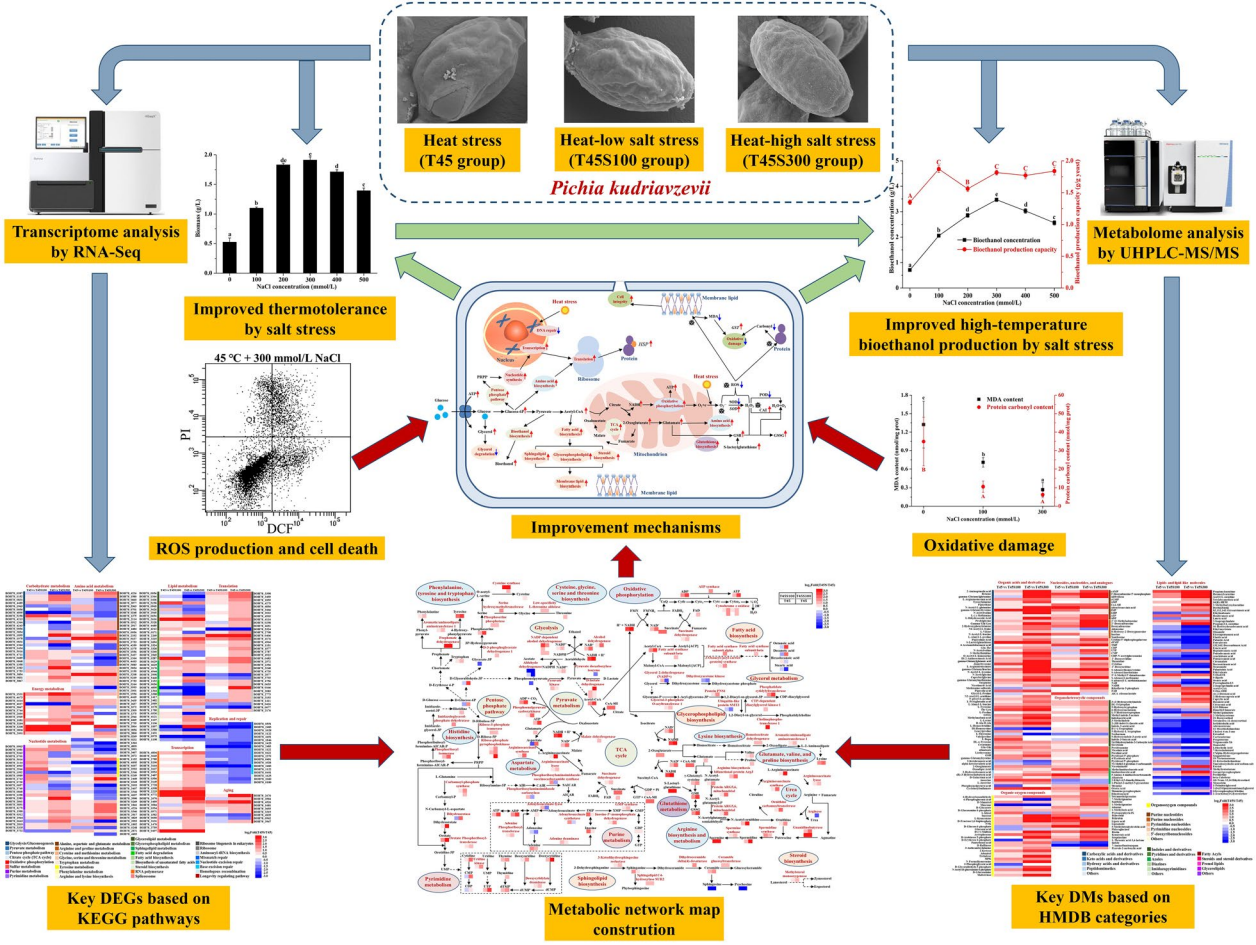

**Supplementary Information:**

The online version contains supplementary material available at 10.1186/s13068-021-02071-0.

## Background

According to the international energy outlook 2020 of the US Energy Information Administration, the world’s energy consumption for transportation will continue to increase at an average annual rate of 1.3% and reach a projected total consumption of roughly 290 exajoule in 2050 [1], resulting in serious environmental pollution if these energy is only provided by fossil fuels. In contrast to fossil fuels, bioethanol is considered as a cleaner alternative due to its high biodegradability, low greenhouse gas emissions up to 96% less than fossil fuels, and null toxicity [2, 3]. In addition, bioethanol is easily miscible with gasoline and can be used as an oxygenated portion in spark-ignition engines to reduce CO_2_ emissions [4]. Compared with other biofuels, such as biodiesel and biohydrogen, bioethanol possesses various advantages, including simple production technology, abundant raw materials and low cost, and has already being produced on an industrial scale in the world. In 2019, the global bioethanol production reached 29 billion gallons, of which the United States and Brazil ranked first and second, respectively, accounting for about 84% of the world’s total bioethanol production [5].

Industrial bioethanol is usually produced by mesophilic yeasts, among which *Saccharomyces cerevisiae* is the most used industrial species with the optimal growth temperatures around 30 °C [6]. However, with the fermentation proceeding, yeasts release heat in the fermentation system, which will reduce the activity of yeasts, eventually leading to the decrease of bioethanol production [7]. To control the fermentation temperature, the cooling equipment with large amount of water and electricity consumption is required, which results in huge fermentation costs. Bioethanol production using thermotolerant yeasts has attracted a growing interest in recent years, which can not only reduce the cooling costs and bacterial contamination [8], but also adapt to the high fermentation temperature (usually over 50 °C) in the simultaneous saccharification and fermentation process [9, 10].

Recently, the yeast *Pichia kudriavzevii* has been used to produce bioethanol at high temperature because of its multi-stress–tolerant characteristics, especially thermotolerance and ethanol tolerance [11–13]. Compared with *S. cerevisiae* strains, *P. kudriavzevii* strains possessed better thermotolerance and bioethanol production over 40 °C [14, 15]. However, the growth of *P. kudriavzevii* strains was obviously suppressed with fermentation temperature rising above 44 °C, resulting in an evident decline of bioethanol production [12, 16]. It is especially necessary to look for the way to improve the thermotolerance of *P. kudriavzevii* strains, since they are applied to high-temperature bioethanol fermentation. Some stress–tolerant microorganisms after being exposed to one stress could cause cross-protection against another different stress, suggesting that there exist similar mechanisms which sense and cause responses to various stresses [17, 18]. It was reported that high concentrations of sugar, such as glucose, maltose, and lactose could significantly improve the thermotolerance of yeasts [19, 20]. Our previous study showed that salt stress induced obvious cross-protection against heat stress of *P. kudriavzevii*, contributing to the improvement of its thermotolerance and bioethanol fermentation [16]. However, the underlying mechanisms of the better thermotolerance and high-temperature bioethanol production of *P. kudriavzevii* by salt stress remain poorly understood.

In this study, the metabolic network construction after comparative transcriptome and metabolome analysis, followed by oxidative stress measurement through flow cytometry and the observations of oxidative damage and antioxidant enzymes, was used to elucidate the underlying mechanisms of the improved thermotolerance and high-temperature bioethanol production of *P. kudriavzevii*. The key genes and metabolic pathways involving in the protective effect of salt stress on heat-induced toxicity in *P. kudriavzevii* were investigated. This study is the first time to report the underlying mechanisms of the improved thermotolerance and high-temperature bioethanol production of *P. kudriavzevii* by salt stress based on cross-protection. The results are expected to develop a novel yeast pretreatment technology for high-temperature bioethanol production. The key genes and metabolic pathways are proposed as useful candidates to construct genetically engineered thermotolerant yeasts for high-temperature bioethanol production in industrial bioprocesses.

## Results

### Effect of salt stress on the thermotolerance and high-temperature bioethanol production

**Fig. 1:**
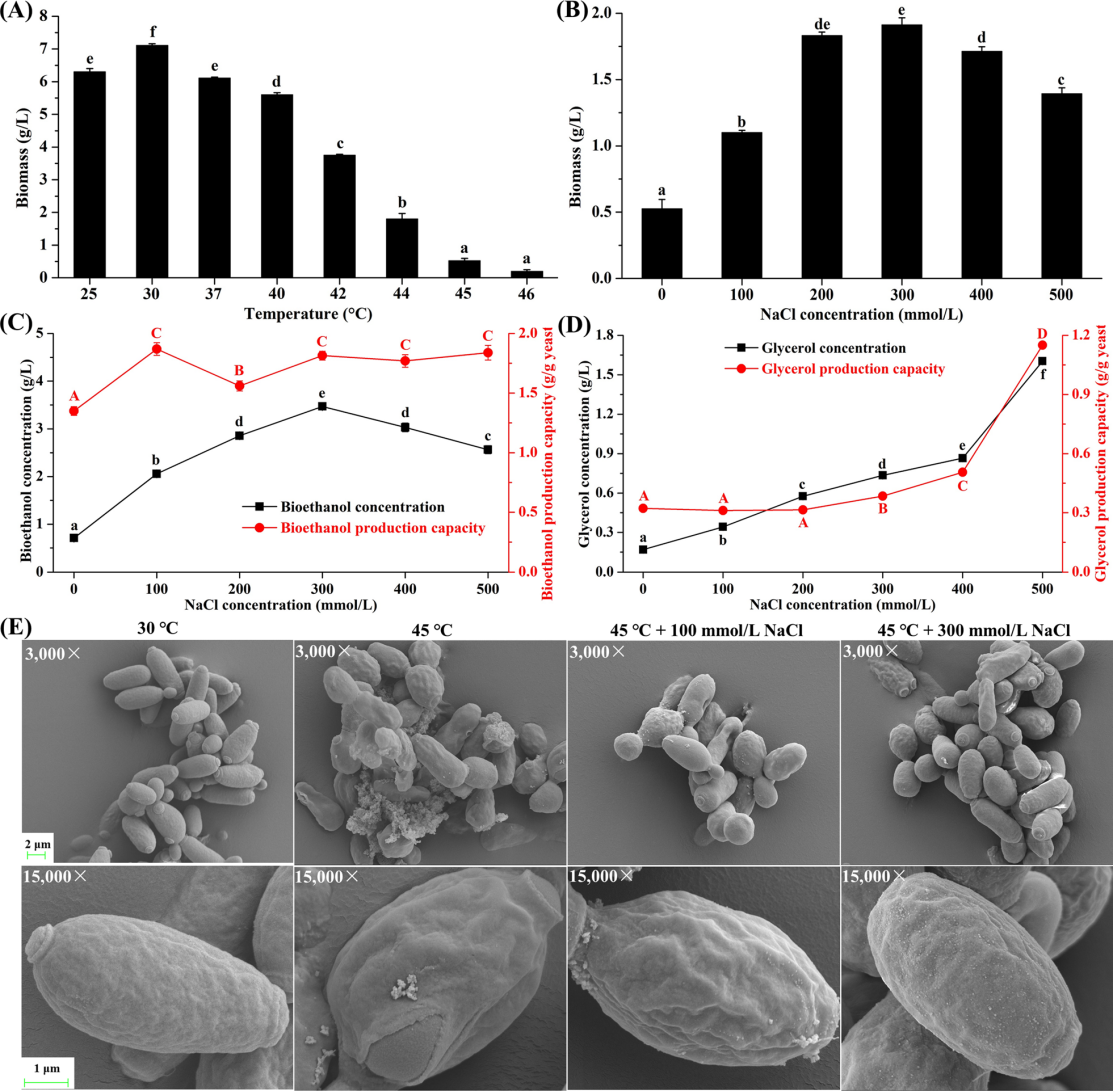
Effect of salt stress on the thermotolerance and high-temperature bioethanol production of *P. kudriavzevii*. **A** Biomass of *P. kudriavzevii* incubated in the liquid YEPD medium for 24 h at various temperatures (30–46 °C). Effect of salt stress (0–500 mmol/L NaCl) on the **B** biomass, **C** bioethanol production, and **D** glycerol production of *P. kudriavzevii* incubated at 45 °C. Data labelled with different letters are statistically different at *p* < 0.05 applying one-way ANOVA and the Tukey test. **E** Effect of salt stress on the cell morphology of *P. kudriavzevii* incubated at 45 °C using SEM

The growth of *P. kudriavzevii* at various temperatures is shown in Fig. 1A. The biomass decreased obviously with the incubation temperature increasing from 30 to 46 °C. As a thermotolerant yeast, *P. kudriavzevii* showed good growth below 44 °C, but was severely suppressed over 45 °C. Interestingly, as shown in Fig. 1B, the addition of NaCl (100–500 mmol/L) caused distinct cross-protection effect against heat stress on *P. kudriavzevii*. The maximum biomass of *P. kudriavzevii* at 45 °C was found at 300 mmol/L NaCl reaching 1.91 g/L, significantly higher than the biomass without NaCl addition (0.53 g/L), and also higher than the biomass at 44 °C (1.80 g/L).

The bioethanol concentrations in the medium incubated with *P. kudriavzevii* at 45 °C were also significantly enhanced by the addition of NaCl (100–500 mmol/L) and reached maximum at 300 mmol/L NaCl, in accordance with the yeast biomass (Fig. 1C). The glycerol concentrations in the medium increased significantly with the increase of NaCl concentrations (0–500 mmol/L) (Fig. 1D). Interestingly, the bioethanol production capacity of *P. kudriavzevii* was significantly improved by all tested salt stress (100–500 mmol/L), while only high salt stress (300–500 mmol/L) significantly enhanced the glycerol production capacity (Fig. 1C, D).

The cross-protection of salt stress on the thermotolerance of *P. kudriavzevii* was further assessed by the cell morphology analysis using scanning electron microscope (SEM) (Fig. 1E). The *P. kudriavzevii* cells exhibited an intact surface morphology at 30 °C. When the incubation temperature increased to 45 °C, the morphology of most cells became irregular, and some of the cells were broken, accompanied by outflow of intracellular materials. Similar to the growth, the cell morphology at 45 °C was obviously improved by the salt stress, especially at 300 mmol/L NaCl. Under this circumstance, the cell morphology was similar to that at 30 °C. Small protrusions appeared on the surface of the cell after salt stress, and the protrusions obviously increased with the increase of salt stress.

### Effect of salt stress on global transcriptome changes under heat stress

Comparative transcriptome analysis based on RNA-Seq was performed to elucidate the underlying mechanisms of the enhanced thermotolerance and high-temperature bioethanol production of *P. kudriavzevii* by salt stress. The total clean bases in each group were over 6.4 Gb with the Q20 and Q30 more than 98% and 93%, respectively (Additional file 1: Table S1). Above 96% of reads were compared to the reference genome of *P. kudriavzevii*, which were sufficient for further analysis. Comparative transcriptome analysis showed that between the T45S100 group and the T45 group there were 374 differentially expressed genes (DEGs), 199 of which were up-regulated, while 175 of which were down-regulated in the T45S100 group (Additional file 1: Fig. S1A). More DEGs (1053 DEGs) were found in the T45 vs T45S300 than in the T45 vs T45S100 (Additional file 1: Fig. S1A). Compared with those in the T45 group, 525 DEGs were up-regulated, while 528 DEGs were down-regulated in the T45S300 group.

Similar main GO terms were found in T45 vs T45S100 and T45 vs T45S300, such as cellular process (162 and 485 DEGs), metabolic process (139 and 388 DEGs), cellular component organization or biogenesis (42 and 160 DEGs) in biological process, cell part (150 and 472 DEGs), membrane part (118 and 297 DEGs), and organelle part (82 and 262 DEGs) in cellular component, as well as catalytic activity (163 and 434 DEGs), binding (144 and 419 DEGs), and transporter activity (35 and 70 DEGs) in molecular function (Additional file 1: Fig. S1B). The DEGs in different comparison groups were then annotated with COG categories (Additional file 1: Fig. S1C). The COG categories in T45 vs T45S100 and T45 vs T45S300 mainly included function unknown (64 and 194 DEGs), posttranslational modification, protein turnover, chaperones (16 and 54 DEGs), amino acid transport and metabolism (22 and 29 DEGs), translation, ribosomal structure and biogenesis (13 and 35 DEGs), transcription (12 and 36 DEGs), as well as carbohydrate transport and metabolism (12 and 24 DEGs). All DEGs in different comparison groups were further annotated with KEGG pathways (Additional file 1: Fig. S1D). The KEGG pathways in T45 vs T45S100 and T45 vs T45S300 were mainly concentrated on the metabolism, such as amino acid metabolism (25 and 49 DEGs), carbohydrate metabolism (20 and 47 DEGs), lipid metabolism (15 and 36 DEGs), metabolism of cofactors and vitamins (16 and 30 DEGs), nucleotide metabolism (13 and 20 DEGs), and energy metabolism (9 and 16 DEGs); genetic information processing, such as translation (14 and 56 DEGs), folding, sorting and degradation (8 and 37 DEGs), transcription (6 and 28 DEGs), and replication and repair (7 and 14 DEGs); cellular processes, such as transport and catabolism (17 and 40 DEGs) and cell growth and death (7 and 28 DEGs); organismal systems, such as aging (3 and 9 DEGs).

**Fig. 2:**
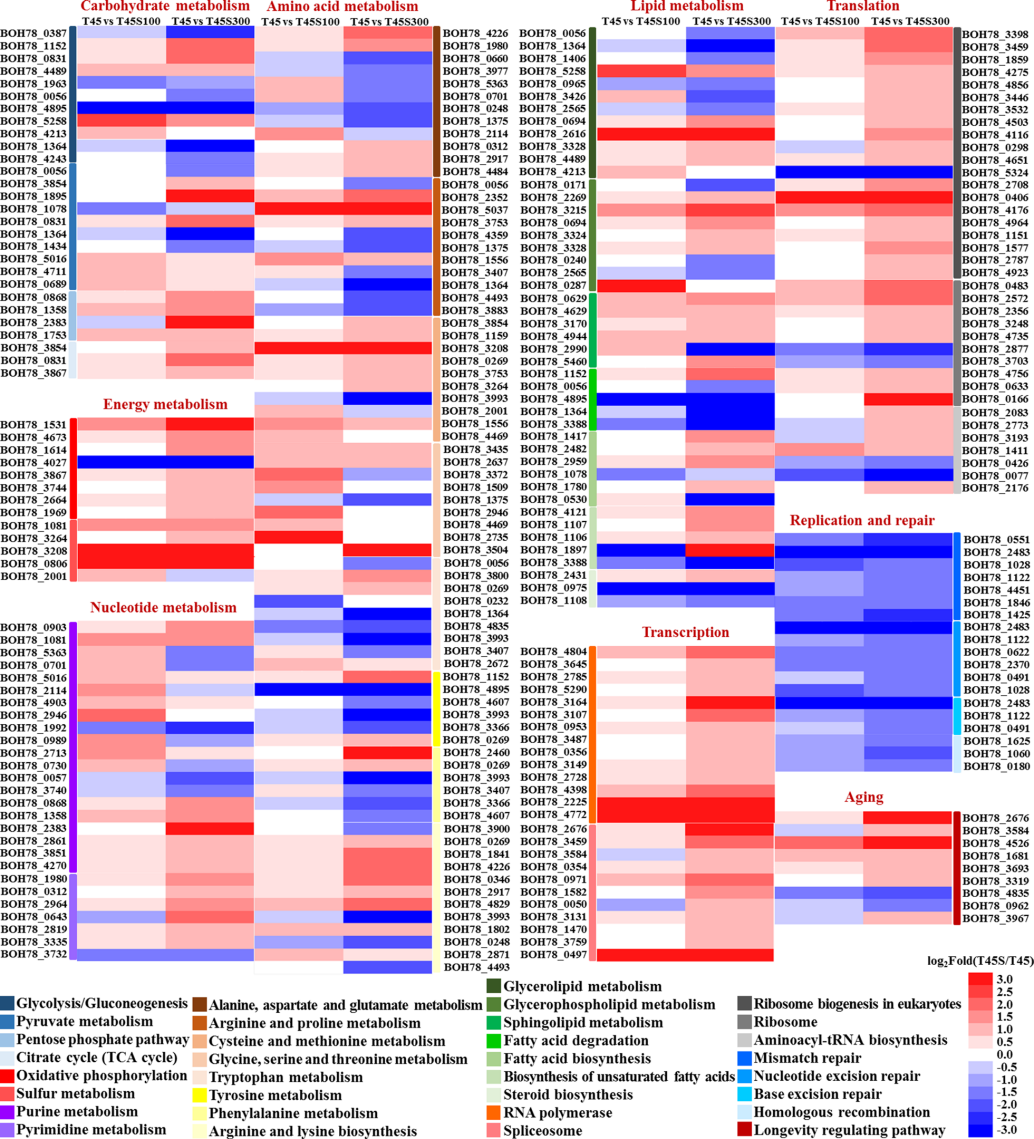
Heatmap profile of DEGs from most annotated KEGG pathways in T45 vs T45S100 and T45 vs T45S300 comparison groups

According to the results of KEGG pathway annotation, the DEGs with the function of metabolism of carbohydrates, energy, nucleotides, amino acids, and lipids were worth noticing (Fig. 2). In carbohydrate metabolism, the DEGs in glycolysis/gluconeogenesis coding for alcohol dehydrogenase (BOH78_1152, BOH78_4489, BOH78_5258, and BOH78_4213) were up-regulated, while the DEGs coding for aldehyde dehydrogenase (BOH78_0056 and BOH78_1364), hexokinase (BOH78_0387 and BOH78_1963), and pyruvate decarboxylase isozyme (BOH78_4243) were down-regulated after treatment with salt stress. In pyruvate metabolism, salt stress positively regulated the expression of genes coding for D-lactate dehydrogenase (BOH78_1895, BOH78_0689, and BOH78_4711) and phosphoenolpyruvate carboxykinase (BOH78_0831) under heat stress. Moreover, the DEGs in pentose phosphate pathway and citrate cycle (TCA cycle) were up-regulated, and higher salt stress correlated to increasing their expression, such as ribose-phosphate pyrophosphokinase (BOH78_0868, BOH78_1358, and BOH78_2383), ribose-5-phosphate isomerase (BOH78_1753), malate dehydrogenase (BOH78_3854), phosphoenolpyruvate carboxykinase (BOH78_0831), and succinate dehydrogenase (BOH78_3867).

Similar to those in carbohydrate metabolism, most DEGs in energy metabolism especially in oxidative phosphorylation and sulfur metabolism were up-regulated by salt stress, such as various subunits of cytochrome c oxidase (BOH78_4673, BOH78_1614, BOH78_2664, and BOH78_1969), ATP synthase (BOH78_3744), sulfate adenylyltransferase (BOH78_1081), cysteine synthase (BOH78_3264 and BOH78_3208), as well as sulfite reductase [NADPH] flavoprotein component (BOH78_0806). For nucleotide metabolism, 8 DEGs in purine metabolism were up-regulated, while 5 DEGs were down-regulated under high salt stress (T45S300 group). More up-regulated DEGs (11 DEGs) and less down-regulated DEGs (1 DEG) were found in T45S100 group than T45S300 group. Similarly, most DEGs in pyrimidine metabolism were up-regulated under salt stress, and higher salt stress enhanced more expression of them. The up-regulated DEGs in nucleotide metabolism mainly included GMP synthase (BOH78_2861), sulfate adenylyltransferase (BOH78_1081), exopolyphosphatase (BOH78_4270), pyruvate kinase (BOH78_5016), carbamoyl-phosphate synthase (BOH78_1980 and BOH78_0312), uridine kinase (BOH78_2964), orotate phosphoribosyltransferase (BOH78_2819), and deoxycytidylate deaminase (BOH78_3335).

Among the KEGG pathways related to metabolism, amino acid metabolism contained the most DEGs. These DEGs were mainly involved in the metabolism of alanine, aspartate, glutamate, arginine, proline, cysteine, methionine, glycine, serine, threonine, tryptophan, tyrosine, and phenylalanine. Interestingly, most DEGs related to these amino acid biosynthesis were up-regulated under salt stress, such as argininosuccinate synthase (BOH78_4226), aromatic/aminoadipate aminotransferase 1 (BOH78_0269), carbamoyl-phosphate synthase (BOH78_1980 and BOH78_0312), argininosuccinate lyase (BOH78_2917), homoisocitrate dehydrogenase, mitochondrial (BOH78_2871), low-specificity L-threonine aldolase (BOH78_3435), phosphoserine phosphatase (BOH78_2637), L-saccharopine oxidase (BOH78_3504), serine hydroxymethyltransferase, cytosolic (BOH78_1509), D-3-phosphoglycerate dehydrogenase 1 (BOH78_4469), adenosylhomocysteinase (BOH78_1159), cysteine synthase (BOH78_3264 and BOH78_3208), spermidine synthase (BOH78_1556 and BOH78_3753), protein ARG5,6, mitochondrial (BOH78_1841), arginine biosynthesis bifunctional protein ArgJ, mitochondrial (BOH78_4829), imidazoleglycerol-phosphate dehydratase (BOH78_4173), and prephenate dehydrogenase [NADP( +)] (BOH78_0416).

For lipid metabolism, a large number of DEGs were involved in the metabolism of glycerolipid, glycerophospholipid and sphingolipid. In these metabolism pathways, the DEGs with function of fatty acid production were up-regulated by salt stress, such as lipase 2 (BOH78_2616) and lysophospholipase 3 (BOH78_2269), while the DEGs responsible for the glycerol degradation were down-regulated including dihydroxyacetone kinase (BOH78_0965) and glycerol 2-dehydrogenase (NADP +) (BOH78_3426). Moreover, most DEGs involved in the biosynthesis of these lipids were improved after treatment with salt stress, such as glycerol-3-phosphate O-acyltransferase 1 (BOH78_3328), phosphatidate cytidylyltransferase (BOH78_3215), CTP-dependent diacylglycerol kinase 1 (BOH78_3324), cholinephosphotransferase 1 (BOH78_0287),sphingolipid C4-hydroxylase SUR2 (BOH78_4629), ceramide glucosyltransferase (BOH78_4944), sphingolipid delta(4)-desaturase (BOH78_5460), and methylsterol monooxygenase (BOH78_2431). In the pathway of fatty acid biosynthesis, most DEGs were up-regulated by salt stress, including 3-oxoacyl-[acyl-carrier-protein] synthase (BOH78_2959) and various subunits of fatty acid synthase (BOH78_1417 and BOH78_2482). Similarly, for biosynthesis of unsaturated fatty acids, most DEGs were up-regulated, including acyl-CoA desaturase (BOH78_4121 and BOH78_1897) and delta(12) fatty acid desaturase (BOH78_1107 and BOH78_1106).

Besides metabolism, the DEGs with the function of genetic information processing were worthy of noting. The DEGs observed in transcription mainly included the pathways of RNA polymerase and spliceosome were significantly up-regulated, and high level of salt stress resulted in the more expression of these DEGs. Similarly, most DEGs in translation were up-regulated by salt stress and were mainly concentrated on the pathways of ribosome biogenesis in eukaryotes, ribosome, and aminoacyl-tRNA biosynthesis. However, for replication and repair, the expression levels of all DEGs in the pathways of mismatch repair, nucleotide excision repair, base excision repair, and homologous recombination were inhibited by salt stress. Moreover, high salt stress enhanced most DEGs in longevity regulating pathway, such as the genes coding for various kinds of superoxide dismutase (BOH78_1681 and BOH78_3319), heat shock protein (BOH78_2676, BOH78_3584, and BOH78_3967), as well as nicotinamidase (BOH78_3693).

### Effect of salt stress on global metabolome changes under heat stress

Comparative metabolome analysis was performed to study the changes of intracellular metabolites of *P. kudriavzevii* under heat stress when treated by salt stress. The principal component analysis (PCA) model was constructed to evaluate discrete differences in global metabolites between different groups (Additional file 1: Fig. S2A). The sum of the first principal component (PC1) and the second principal component (PC2) was 88.8% of the total variation. In these 2D-PCA score plots, the T45 group showed more similarity with the T45S100 group than T45S300 group. Comparative metabolome analysis showed that there was a total of 457 differential metabolites (DMs) between the T45S100 group and the T45 group in ESI + /ESI−, among which 309 DMs were improved by salt stress, while 148 DMs were inhibited (Additional file 1: Fig. S2B). More DMs (684 DMs) were found in the T45 vs T45S300 than T45 vs T45S100 (Additional file 1: Fig. S2B). Compared with those in the T45 group, the contents of 433 DMs increased, while the contents of 251 DMs decreased in the T45S300 group. There were 323 common DMs in the T45 vs T45S100 and T45 vs T45S300, and the unique DMs increased with the increase of salt stress (Additional file 1: Fig. S2C).

The DMs in different comparison groups were annotated with HMDB categories (Additional file 1: Fig. S2D). The main HMDB categories were similar in T45 vs T45S100 and T45 vs T45S300, mainly including organic acids and derivatives (37 and 60 DMs), lipids and lipid-like molecules (35 and 59 DMs), organoheterocyclic compounds (34 and 44 DMs), nucleosides, nucleotides, and analogues (19 and 30 DMs), as well as organic oxygen compounds (16 and 24 DMs). All DMs in different comparison groups were further mapped to KEGG pathways (Additional file 1: Fig. S2E). The KEGG pathways in T45 vs T45S100 and T45 vs T45S300 were mainly concentrated on the metabolism of carbohydrates, amino acids, and lipids, such as the tryptophan metabolism (4 and 10 DMs), fatty acid biosynthesis (4 and 5 DMs), beta-alanine metabolism (4 and 6 DMs), fatty acid degradation (3 and 3 DMs), as well as amino sugar and nucleotide sugar metabolism (3 and 3 DMs).

**Fig. 3:**
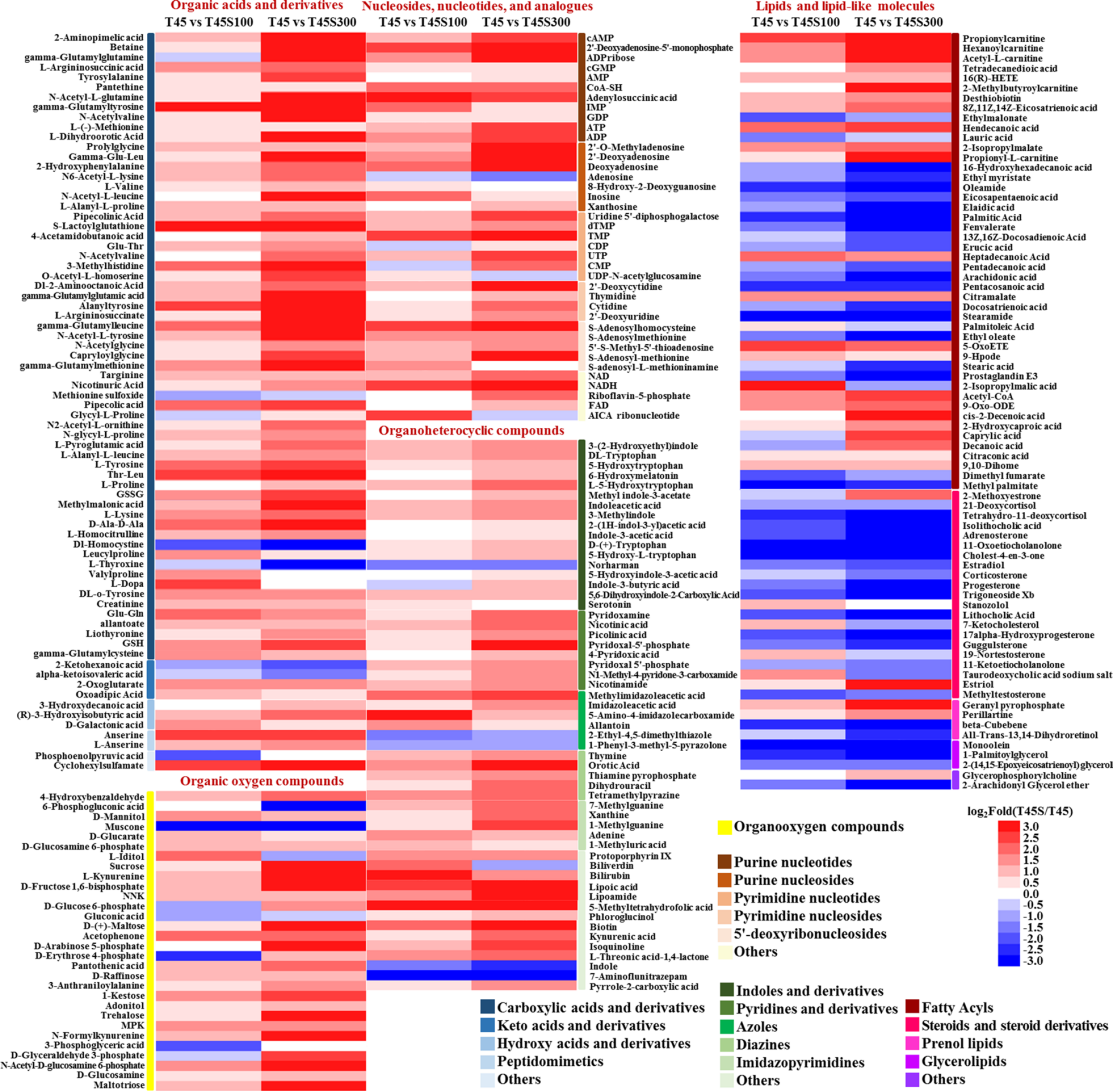
Heatmap profile of DMs from most annotated HMDB categories in T45 vs T45S100 and T45 vs T45S300 comparison groups

According to the results of HMDB category annotation, the DMs that were annotated as organic acids and derivatives were worth noticing, among which carboxylic acids and derivatives were the main DMs (Fig. 3). Interestingly, most DMs involving in carboxylic acids and derivatives were enhanced by salt stress, and higher salt stress improved their content, mainly including various kinds of amino acids, peptides, and analogues. In addition, many DMs belonging to keto acids, hydroxy acids, and peptidomimetics were also up-regulated by salt stress. Similar to carboxylic acids and derivatives, most DMs belonging as organooxygen compounds increased with the increase of salt stress. Especially, many carbohydrates and carbohydrate conjugates in *P. kudriavzevii* in the T45S300 group, such as sucrose, d-( +)-maltose, d-raffinose, 1-kestose, trehalose, maltotriose, d-fructose 1,6-bisphosphate, d-glucose 6-phosphate, d-arabinose 5-phosphate, d-glyceraldehyde 3-phosphate, *N*-acetyl-d-glucosamine 6-phosphate, and d-glucosamine were significantly higher than those in the T45 group. Among the DMs in nucleosides, nucleotides, and analogues, most of them were annotated as purine nucleotides, purine nucleosides, pyrimidine nucleotides, pyrimidine nucleosides, and 5'-deoxyribonucleosides were significantly improved under salt stress. In addition, salt stress also markedly enhanced the contents of dinucleotides including NAD and NADH. Similarly, most DMs in organoheterocyclic compounds were improved by salt stress, which were mainly classified as indoles and derivatives, pyridines and derivatives, azoles, diazines, imidazopyrimidines, dithiolanes, pteridines and derivatives, as well as quinolines and derivatives. Although most DMs in *P. kudriavzevii* were enhanced by salt stress, many DMs belonging to lipids and lipid-like molecules were inhibited, such as fatty acyls, steroids and steroid derivatives, prenol lipids, and glycerolipids. However, there were exceptions that some fatty acyl CoAs such as acetyl-CoA and some glycerophospholipids such as glycerophosphorylcholine, and many unsaturated fatty acids were improved by salt stress especially in T45S300 group.

### Effect of salt stress on heat-induced oxidative stress

**Fig. 4:**
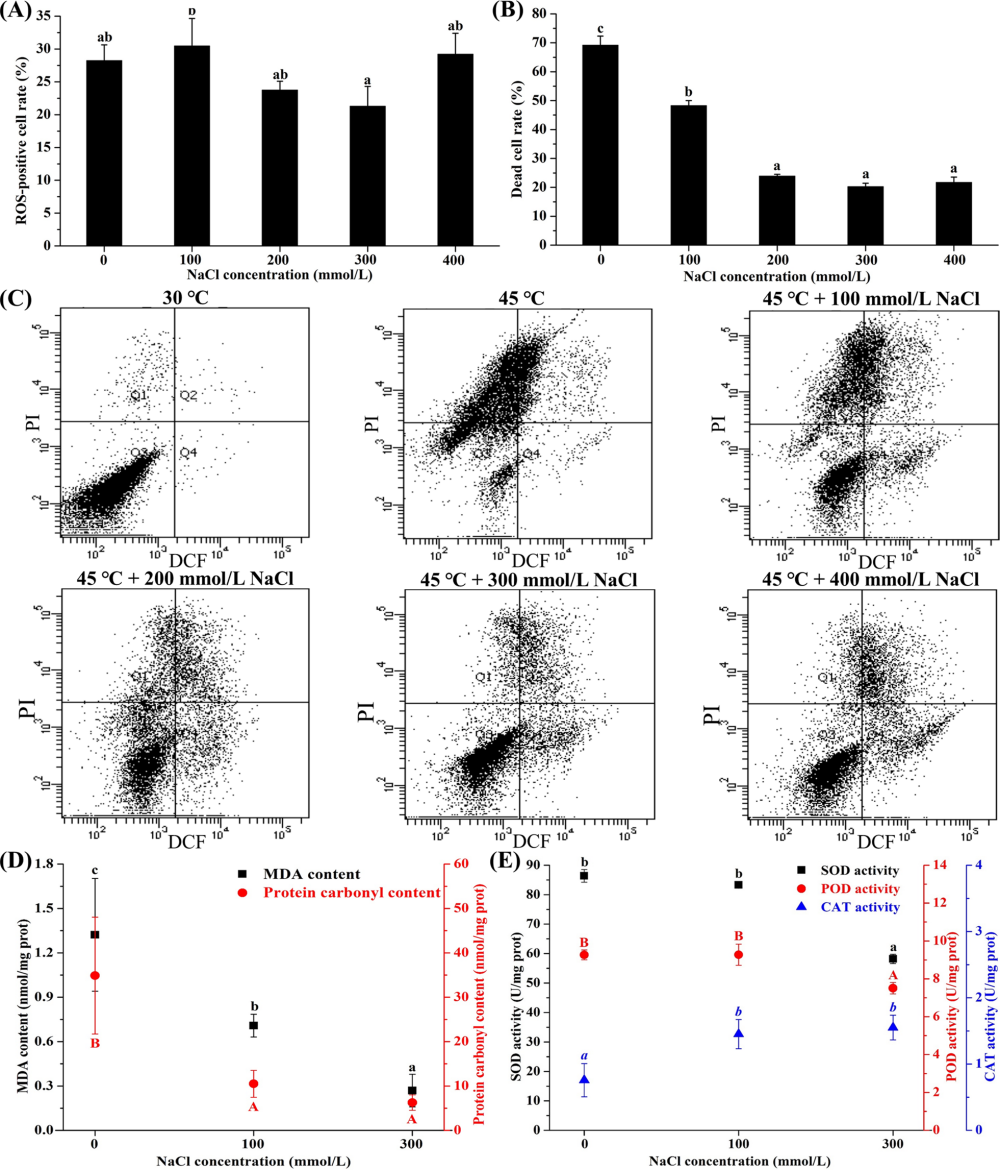
Effect of salt stress on the heat-induced oxidative stress in *P. kudriavzevii*. Effect of salt stress on the **A** ROS-positive cell rate and **B** dead cell rate of *P. kudriavzevii* incubated at 45 °C. **C** Representative flow cytometry dot plots after double staining with DCFH-DA and PI. Q1: DCF^−^/PI^+^, ROS-negative cells/dead cells; Q2: DCF^+^/PI^+^, ROS-positive cells/dead cells; Q3: DCF^−^/PI^−^, ROS-negative cells/living cells; Q4: DCF^+^/PI^−^, ROS-positive cells/living cells. Effects of salt stress on the **D** oxidative damage and **E** antioxidant enzyme activities of *P. kudriavzevii* incubated at 45 °C. Data labelled with different letters are statistically different at *p* < 0.05 applying one-way ANOVA and the Tukey test

The effect of salt stress on the heat-induced reactive oxygen species (ROS) production, cell death, oxidative damage, as well as antioxidant enzyme activities was studied in *P. kudriavzevii* (Fig. 4). Heat induced obvious ROS production and cell death in *P. kudriavzevii*. The ROS-positive cell rate and dead cell rate, respectively, rose from 0.5% and 2.4% to 28.2% and 69.2%, with the incubation temperature increasing from 30 to 45 °C (Fig. 4A–C). Interestingly, the heat-induced ROS production and cell death significantly decreased under salt stress. With the increase of NaCl concentrations, the ROS-positive cell rate first decreased and increased, while the dead cell rate sharply decreased. The maximum inhibition of ROS production and cell death was observed at 300 mmol/L NaCl, which was consistent with the results of biomass. Under this level of salt stress, the ROS-positive cell rate and dead cell rate, respectively, fell to only 21.3% and 20.3%. Although the ROS-positive cell rate did not decline as much as dead cell rate after salt addition, the ROS-positive cells under salt stress were mainly concentrated in the living cells, whereas they were almost dead cells under only heat stress. Malonaldehyde (MDA) and protein carbonyl are, respectively, used to assess the oxidative damage of membrane lipids and proteins [21]. In this study, similar to ROS production and cell death, the heat-induced oxidative damage of membrane lipids and proteins were obviously attenuated by salt stress, especially at 300 mmol/L NaCl (Fig. 4D). Different effects of salt stress on the activities of different antioxidant enzymes under heat stress were observed (Fig. 4E). The superoxide dismutase (SOD) and peroxidase (POD) activities under heat stress were not significantly affected by NaCl at 100 mmol/L, but were significantly reduced at 300 mmol/L. The catalase (CAT) activity was markedly improved by salt stress, and much higher CAT activity was observed at 300 mmol/L NaCl.

## Discussion

**Fig. 5:**
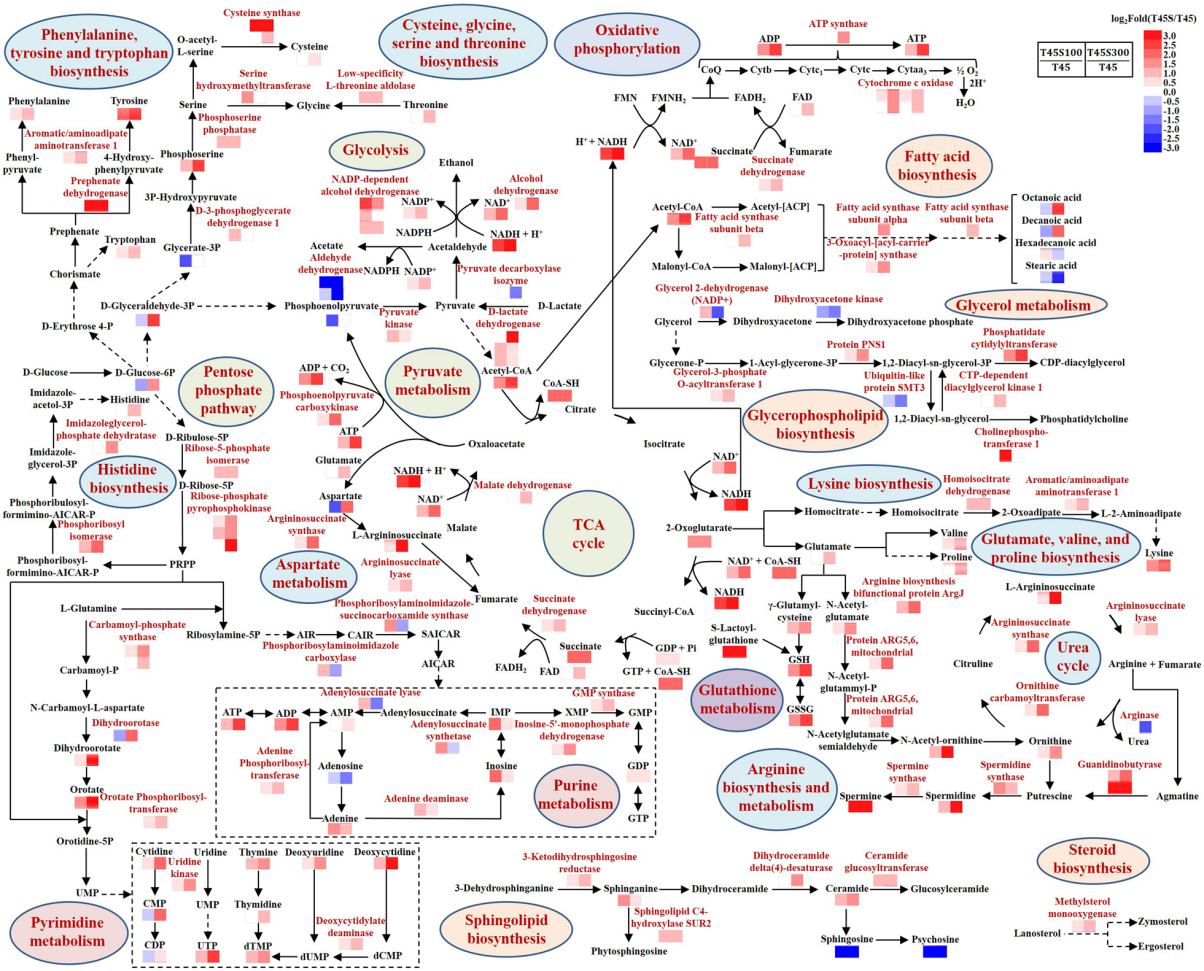
Construction of metabolic network map of *P. kudriavzevii* based on the data of combined analysis of comparative transcriptome and metabolome. The key enzymes and metabolites represented in the metabolic network map were, respectively, from the heatmaps of the DEGs and DMs

**Fig. 6:**
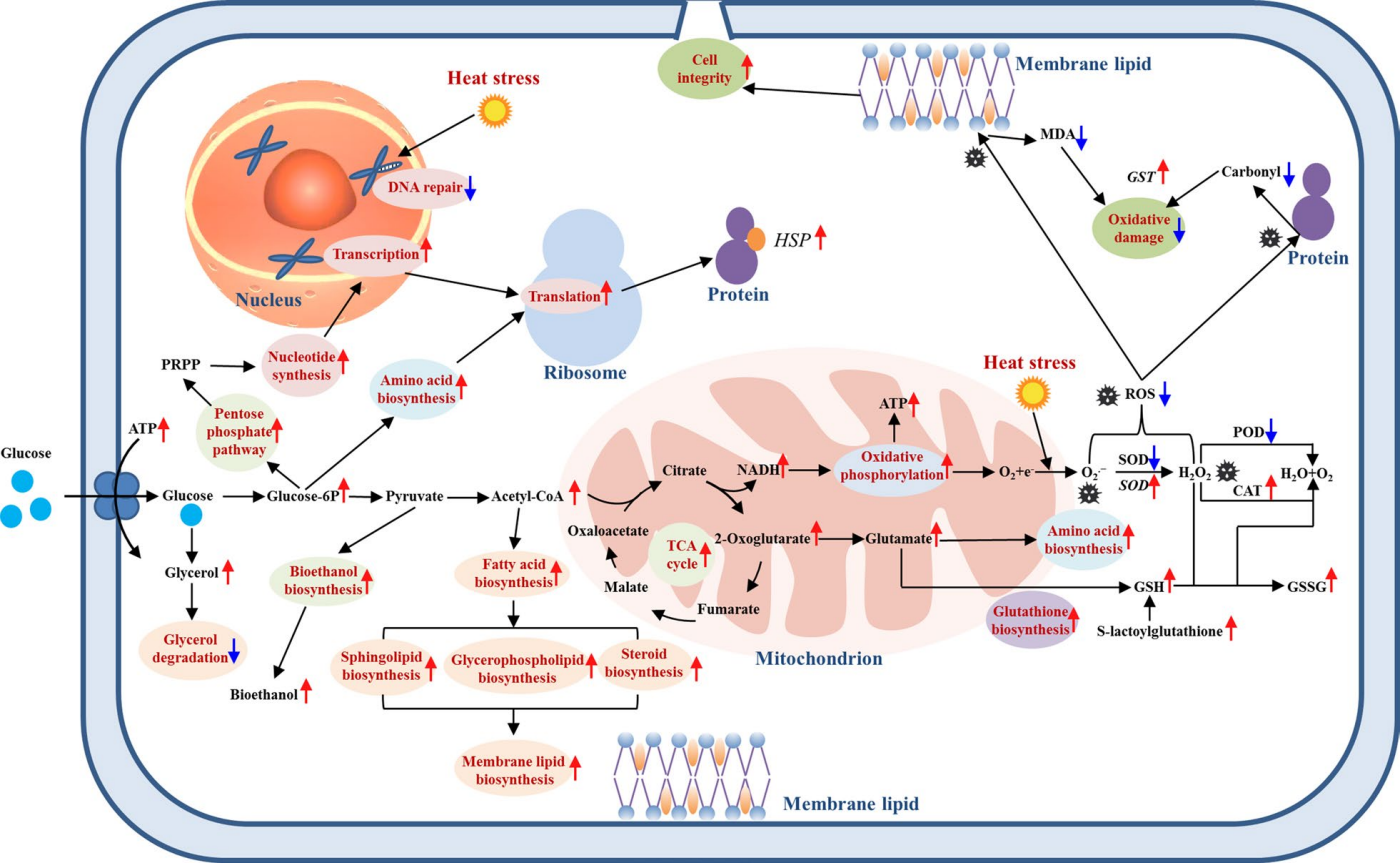
Schematic representation of the mechanisms of improved thermotolerance of *P. kudriavzevii* by salt stress. Red and blue arrows, respectively, indicate the improved and reduced metabolic process, gene expression, substance content, or enzyme activity in *P. kudriavzevii* under heat stress by salt stress

The thermotolerance of yeasts plays a key role in their bioethanol production at high temperature. Although *P. kudriavzevii* possessed good thermotolerance, its growth was severely inhibited over 45 °C. Interestingly, the heat-induced inhibition effect on *P. kudriavzevii* was significantly ameliorated by a combined stress of salt, contributing to its high-temperature bioethanol production (Fig. 1). According to the results of comparative transcriptome and metabolome analysis, the cell metabolisms under heat stress including the metabolisms of carbohydrates, energy, amino acids, nucleotides, and lipids were obviously changed. To better understand the relationship among the metabolism changes, thermotolerance, and bioethanol production, the metabolic network map including major metabolic pathways in *P. kudriavzevii* were constructed (Fig. 5). The key genes and metabolites related to the improvement of thermotolerance and high-temperature bioethanol production of *P. kudriavzevii* by salt stress were obtained (Additional file 1: Tables S2 and S3). Based on the above results, the mechanisms of improved thermotolerance and high-temperature bioethanol production of *P. kudriavzevii* by salt stress were summarized (Fig. 6).

Carbohydrate metabolism and energy metabolism provide necessary energy for the normal growth and reproduction of yeasts. In the present study, more glucose-6P and glyceraldehyde-3P were produced after 300 mmol/L NaCl addition, and were helpful for the catalytic reactions in pentose phosphate pathway and glycolysis. Pyruvate and acetyl-CoA are important intermediate compounds in the metabolism of carbohydrates. In this study, the enhanced expression of pyruvate kinase (BOH78_5016) and D-lactate dehydrogenase (BOH78_0689) by salt stress contributed to the formation of pyruvate. The increase of acetyl-CoA in both salt-treated groups stimulated most catalytic reactions in the TCA cycle, leading to the increasing NADH, which entered the respiratory chain in the mitochondria for ATP synthesis. Previous studies showed that ATP levels in yeast cells decreased significantly under heat stress, and the ATP synthesis increased along with the improved thermotolerance by sugar treatment [19, 20]. Similar results were found in this study that the ATP content under heat stress was significantly enhanced by salt stress through the up-regulated cytochrome c oxidase (BOH78_4673, BOH78_1614, BOH78_2664, and BOH78_1969) and ATP synthase (BOH78_3744), contributing to the thermotolerance of *P. kudriavzevii*.

Carbohydrate metabolism is not only essential for energy production, but also provides crucial intermediates for the conversion of other nutrients. The improved 2-oxoglutarate by salt stress contributed to the increase of intracellular glutamate, valine, proline, lysine and ornithine in *P. kudriavzevii* under heat stress. The increase of glucose-6P after 300 mmol/L NaCl addition played an important role in the promoting the amino acid biosynthesis, including phenylalanine, tyrosine, tryptophan, cysteine, glycine, serine, and threonine. Meanwhile, the up-regulated ribose-5-phosphate isomerase (BOH78_1753) and ribose-phosphate pyrophosphokinase (BOH78_0868, BOH78_1358, and BOH78_2383) facilitated the conversion from D-ribulose-5P to phosphoribosyl pyrophosphate (PRPP), which was helpful to the purine and pyrimidine biosynthesis under heat stress. Interestingly, most genes related to transcription including RNA polymerase and spliceosome, as well as translation including ribosome biogenesis, ribosome, and aminoacyl-tRNA biosynthesis were up-regulated by salt stress especially in the T45S300 group, consistent with the changes of most amino acids and nucleotides. Ribosome biogenesis in the nucleolus under heat stress accelerates to support the production of proteins for heat response [22]. The rRNA-processing protein CgrA played an important role in the thermotolerance of *Aspergillus fumigatus* [23]. In this study, the genes coding for rRNA-processing protein RRP7 (BOH78_3446) and FCF1 (BOH78_0406) were all up-regulated by salt stress, probably contributing to the improvement of thermotolerance of *P. kudriavzevii*.

Under heat stress, yeasts tend to convert glucose to trehalose, which can help to form a hydration shell around the cell membrane by forming hydrogen bonds with phospholipids to protect against heat damage [24]. Trehalose can also prevent intracellular proteins from denaturing through the formation of hydration shell [25]. In this study, the intracellular trehalose under heat stress was significantly improved by salt stress, especially in the T45S300 group, in which the trehalose concentration increased by 13.4 times than that in the T45 group. The abundant trehalose production contributed to the cross-protection of salt stress for the thermotolerance of *P. kudriavzevii*. Glycerol overproduction helps yeast cells cope with the adverse effects of heat stress, such as protecting proteins from thermal denaturation [26]. Faster glycerol production was found in thermotolerant yeast strains than thermosensitive strains [27]. In this study, salt stress significantly enhanced the glycerol production capacity of *P. kudriavzevii* under heat stress (Fig. 1D). Meanwhile, the expression levels of genes related to glycerol degradation including glycerol 2-dehydrogenase (NADP +) (BOH78_3426) and dihydroxyacetone kinase (BOH78_0965) in the T45S300 group were, respectively, reduced to 29.4% and 39.3% of those in the T45 group. The enhanced glycerol production and inhibited glycerol degradation by salt stress might played an important role in the thermotolerance improvement of *P. kudriavzevii*.

Heat shock proteins (HSPs) play a key role to maintain the normal functions of cells upon heat stress. Their main function is to prevent and repair denatured or misfolded proteins induced by heat [24]. In this study, the heat shock protein 78 (BOH78_3967), SSA2 (BOH78_3584), and SSA3 (BOH78_2676) were significantly up-regulated in the T45S300 group (Fig. 2), contributing to the better thermotolerance of *P. kudriavzevii*. Similarly, previous study showed that salt stress positively regulated the expression of various HSPs in *P. kudriavzevii* under cadmium stress [28]. These results suggested that HSPs in *P. kudriavzevii* were easily induced by salt stress, which could help cells survive under other extreme stresses, such as heat and cadmium.

Yeasts maintain membrane fluidity by generating CoA-activated unsaturated fatty acids (UFAs) as lipid building blocks using delta-9 desaturase [29]. It was reported that the overexpression of delta-9 desaturase gene *OLE1* increased fatty acid unsaturation, helping to counter heat-induced lipid peroxidation in *S. cerevisiae* [2]. Similarly, the delta-9 desaturase gene *OLE1* of *P. kudriavzevii* (BOH78_4121 and BOH78_1897) was significantly up-regulated by salt stress (Fig. 2), in accordance with the decrease of lipid peroxidation (Fig. 4D) and improvement of thermotolerance (Fig. 1). Salt stress also induced the expression of the genes coding for delta(12) fatty acid desaturase FAD2 (BOH78_1107 and BOH78_1106) (Fig. 2), which could introduce a double bond in the fatty acid chain three carbons away from an existing double bond to biosynthesize polyunsaturated fatty acids (PUFAs) endogenously. Meanwhile, the up-regulated genes involving in UFA biosynthesis led to the increase of UFAs, such as 16(R)-HETE, 8Z,11Z,14Z-eicosatrienoic acid, 5-oxoETE, 9-hpode, 9-oxo-ODE, cis-2-decenoic acid, and 9,10-dihome in *P. kudriavzevii* (Fig. 3), contributing to the stability of membrane structure under heat stress. Ergosterol, as an important yeast sterol, takes part in maintaining the fluidity, integrity and permeability of cell membrane [30]. The enhanced ergosterol production obviously improved the thermotolerance of *S. pombe* [31]. In this study, salt stress markedly promoted the expression of methylsterol monooxygenase (BOH78_2431) which participated in ergosterol biosynthesis. Phytosphingosine and glucosylceramide are important sphingolipids responsible for membrane lipid biosynthesis. The enzymes that catalyzed their biosynthesis, such as 3-ketodihydrosphingosine reductase (BOH78_3170), sphingolipid delta(4)-desaturase (BOH78_5460), dihydroceramide delta(4)-desaturase (BOH78_5460), and ceramide glucosyltransferase (BOH78_4944), as well as the intermediate products for synthesizing them including sphinganine and ceramide, were all significantly enhanced by salt stress. The pathway related to the biosynthesis of glycerophospholipids such as CDP-diacylglycerol and phosphatidylcholine were also stimulated by salt stress, in which the improved enzymes including glycerol-3-phosphate O-acyltransferase 1 (BOH78_3328), protein PNS1 (BOH78_0694), phosphatidate cytidylyltransferase (BOH78_3215), CTP-dependent diacylglycerol kinase 1 (BOH78_3324), and cholinephosphotransferase 1 (BOH78_0287) played the key roles. The improved biosynthesis of steroid, sphingolipid, and glycerophospholipid by salt stress helped to maintain the stability and normality of membrane lipids under heat stress.

Heat stress can induce the ROS outburst in cells, causing pleiotropic damage of biomolecules which may result in cell death [26]. In this study, obvious heat-induced ROS generation was also found in *P. kudriavzevii* (Fig. 4A), along with severe oxidative damage of proteins and membrane lipids (Fig. 4D), ultimately leading to cell breakage (Fig. 1F) and cell death (Fig. 4B). However, the heat-induced oxidative damage and cell death were obviously alleviated by salt stress (Fig. 4). To mitigate the oxidative damage induced by ROS, yeasts have developed a complex antioxidative defense system, including antioxidant enzymes and non-enzyme scavengers. Our previous study showed that salt stress significantly improved the gene expression of antioxidant enzymes and their activities (SOD, CAT and POD) in *P. kudriavzevii* under cadmium stress, which inhibited the oxidative damage and improved the cadmium tolerance [28]. In this study, the genes coding for SOD including SOD-Fe (BOH78_3319) and SOD-Mn (BOH78_1681) were obviously improved by salt stress (Fig. 2). However, the SOD activity was lower than that under heat stress alone (Fig. 4E). Many studies have proved the important role of CAT activity on the yeast thermotolerance [19, 32]. Similarly, the CAT activity under heat stress in *P. kudriavzevii* was ameliorated by salt stress (Fig. 4E), contributing to the scavenging of H_2_O_2_. As an important non-enzyme scavenger, glutathione (GSH) can protect cells from oxidative stress by clearing H_2_O_2_ [18]. It was reported that GSH could help *Saccharomyces cerevisiae* strains survive at higher temperatures [33]. In this study, the intracellular GSH of *P. kudriavzevii* under heat stress was, respectively, enhanced by 2.2 and 4.8 times after addition of 100 and 300 mmol/L NaCl, while its oxidation product glutathione disulfide (GSSG), respectively, increased by 1.8 and 4.3 times. Moreover, salt stress significantly improved its precursor compounds, among which γ-glutamylcysteine increased by 2.1 and 1.2 times, while S-lactoylglutathione increased by 19.6 and 83.6 times after addition of 100 and 300 mmol/L NaCl, respectively. Glutathione S-transferases (GSTs) can catalyze the reaction of GSH with oxidative damage by-products of biomolecules to minimize the oxidative damage and prooxidant-induced cell death [34, 35]. In this study, the gene coding for glutathione S-transferase Y-2 (BOH78_1912) was significantly up-regulated by salt stress, especially at 300 mmol/L NaCl with the improved expression by 76.1 times. Interestingly, the up-regulated expression of this gene also played an important role in the reduction of cadmium-induced oxidative damage in *P. kudriavzevii* by salt stress [28] and acid stress [18]. The improved antioxidant defense system by salt stress in *P. kudriavzevii*, expecially the involvement of GSH and GST, significantly reduced the heat-induced ROS outburst, oxidative damage, and cell death, which was another crucial mechanism of the improved thermotolerance.

The bioethanol fermentation ability of yeasts at high temperature is closely related to its thermotolerance. This study showed that the total bioethanol production in the medium was significantly enhanced by salt stress, mainly resulting from the improvement of thermotolerance (Fig. 1C). Moreover, the bioethanol production capacity per unit of *P. kudriavzevii* biomass was also improved by salt stress (Fig. 1C). The metabolic network map showed that salt stress stimulated the carbohydrate metabolism, which provided abundant intermediates for the conversion of bioethanol. Meanwhile, the expression levels of alcohol dehydrogenases (BOH78_1152, BOH78_5258, BOH78_4213, and BOH78_4489) in the *P. kudriavzevii* which could catalyze the conversion of acetaldehyde to bioethanol were significantly improved by salt stress. Salt stress also obviously inhibited the expression of aldehyde dehydrogenases (BOH78_0056 and BOH78_1364), which reduced the conversion of acetaldehyde to acetic acid and provided more acetaldehyde to produce bioethanol. These results indicated that the improvement of bioethanol production was not only due to the improved thermotolerance by salt stress, but also resulted from the regulation of enzymes for the production of more bioethanol, including the up-regulated alcohol dehydrogenases and down-regulated aldehyde dehydrogenases. This study proved that salt stress was an effective method to improve the high-temperature bioethanol production of *P. kudriavzevii*.

## Conclusion

Salt stress significantly improved the thermotolerance and high-temperature bioethanol production of *P. kudriavzevii* based on cross-protection. Metabolic network map showed that the enzymes and intermediates in carbohydrate metabolism were significantly improved by salt stress, contributing to bioethanol synthesis and intracellular metabolisms. The increasing trehalose, glycerol, HSPs, and ergosterol helped maintain the cell component stability under heat stress. Salt stress stimulated the antioxidant defense system, expecially GSH and GST, leading to a sharp decline in heat-induced ROS outburst, oxidative damage, and cell death. The up-regulated alcohol dehydrogenases and down-regulated aldehyde dehydrogenases by salt stress played another key role in the improved bioethanol production. Overall, this work provides a novel insight of developing pretreatment technology using salt stress to improve the thermotolerance and high-temperature bioethanol production of *P. kudriavzevii*. The key genes and metabolic pathways are also helpful in constructing genetically engineered thermotolerant yeasts for high-temperature bioethanol production.

## Materials and methods

### Yeast activation

*P. kudriavzevii* A16 was isolated from a starter of Chinese high-temperature fermentation Baijiu [36]. The yeast activation was done as follows. The preserved yeast was first incubated on YEPD agar slant (10 g/L yeast extract powder, 20 g/L peptone, 20 g/L glucose, and 20 g/L agar; pH 5.0). After incubation for 24 h at 30 °C, the yeast cells in the slant were then incubated in liquid YEPD medium (10 g/L yeast extract powder, 20 g/L peptone, and 20 g/L glucose; pH 5.0) for 24 h at 30 °C and 180 r/min to keep them activated in the environment of liquid YEPD medium using for follow-up experiments [18].

### Effect of salt stress on the growth and bioethanol production under heat stress

Yeast cells after activation at initial biomass of 0.1 g/L were incubated at various temperatures (30, 37, 40, 42, 44, 45, and 46 °C) to evaluate its thermotolerance. The influence of salt stress on the yeast growth under heat stress was studied by adding various concentrations of NaCl (0, 100, 200, 300, 400, and 500 mmol/L) to the liquid medium and then incubating for 24 h at 45 °C. The yeast cells after incubation were centrifuged, washed with pure water, and weighed after being dried to constant weight at 105 °C. The biomass (g/L) was calculated to analyze the yeast thermotolerance under various levels of salt stress. The bioethanol and glycerol concentrations (g/L) in the medium were analyzed by the ACQUITY UPLC H-Class System (Waters Corp., USA) using the ICSep Coregel 87H3 column (300 × 7.8 mm; Transgenomic Inc., USA) as described previously [16]. The bioethanol production capacity (g/g yeast) and glycerol production capacity (g/g yeast) of *P. kudriavzevii* were calculated according to their concentrations in the medium and yeast biomass.

### Cell morphology observation by scanning electron microscope

*P. kudriavzevii* cells after activation were incubated for 24 h at 45 °C with different concentrations of NaCl (0, 100, and 300 mmol/L). The yeast incubating for 24 h at 30 °C was used as control. After centrifugation at 8000 r/min and 4 °C for 5 min, the *P. kudriavzevii* cells were fixed with 2.5% glutaraldehyde for 4 h, and were then washed three times with phosphate-buffered saline (50 mmol/L, pH 7.0). The dehydration process was used as follows: 30% ethanol, 15 min; 50% ethanol, 15 min; 70% ethanol, 15 min; 85% ethanol, 15 min; 90% ethanol, 15 min; 100% ethanol, 15 min, twice. After centrifugation at 8000 r/min and 4 °C for 5 min, the cells were, respectively, frozen at − 20 °C, − 40 °C, and − 80 °C for 12 h in order. After being lyophilized and sputtered with gold, the samples were used for cell morphology observation by the MERLIN field emission scanning electron microscope (ZEISS, Germany) [37].

### Transcriptome analysis by RNA-Seq

*P. kudriavzevii* cells after activation were, respectively, incubated (1) at 45 °C (T45 group), at 45 °C with 100 mmol/L NaCl (T45S100 group), and (3) at 45 °C with 300 mmol/L NaCl (T45S300 group) in liquid YEPD medium. Transcriptome analysis by RNA-Seq was performed as described previously [28] with some modifications. Briefly, after incubation for 24 h, the cells were centrifuged for the total RNA isolation using Trizol Reagent (Invitrogen, USA). RNA-Seq library was prepared by TruSeq™ RNA sample preparation Kit (Illumina, USA) using 1.0 μg of total RNA. The mRNAs were isolated according to polyA selection method by oligo(dT) beads, and were then fragmented for the construction of cDNA libraries. After purification, the cDNA libraries were sequenced on the HiSeq xten sequencer (Illumina, USA) in the form of paired-end sequencing (2 × 150 bp read length). The sequencing reads were compared to the genome of *P. kudriavzevii* 129 using TopHat (v2.1.1). The gene expression in each group was calculated according to the fragments per kilobase of exon per million mapped reads method.

### Metabolome analysis by UHPLC–MS/MS

*P. kudriavzevii* cells in different groups were resuspended with prechilled 80% methanol by well vortex, and were placed in liquid nitrogen for 5 min. The samples were melted on ice, and then were whirled for 30 s. After sonification for 6 min, they were centrifuged at 5000 r/min and 4 °C for 1 min. The supernatant was lyophilized and dissolved with 10% methanol, which was used for UHPLC–MS/MS analysis by the Vanquish UHPLC system (ThermoFisher, Germany) equipped with the Orbitrap Q Exactive™ HF mass spectrometer (Thermo Fisher, Germany) in ESI + /ESI − modes as described previously [38]. Briefly, The samples were injected onto a Hypesil Gold column (100 × 2.1 mm, 1.9 μm) using a 17 min linear gradient at a flow rate of 0.2 mL/min. The eluents for ESI + mode were eluent A (0.1% formic acid in water) and eluent B (methanol). The eluents for ESI- mode were eluent A (5 mmol/L ammonium acetate; pH 9.0) and eluent B (methanol). The linear gradient elution program was used as follows: 2% B, 1.5 min; 2–100% B, 12.0 min; 100% B, 14.0 min; 100–2% B, 14.1 min; 2% B, 17 min. The Q Exactive™ HF mass spectrometer was operated in ESI + /ESI- with spray voltage of 3.2 kV, capillary temperature of 320 °C, sheath gas flow rate of 40 arb, and aux gas flow rate of 10 arb.

The raw data from UHPLC–MS/MS were treated by the Compound Discoverer (v3.1) to perform the peak alignment, peak picking, and metabolite quantitation. After the peak intensities were normalized to the total spectral intensity, the data were used to predict the molecular formula according to the additive ions, molecular ion peaks, and fragment ions. Then the peaks were matched with the mzVault, mzCloud, and MassList database to get their accurate qualitative and relative quantitative results.

### Determination of oxidative stress and antioxidant enzyme activity

Yeast cells in different groups were used to determine the oxidative stress including ROS, MDA, protein carbonyls, and cell death, as well as antioxidant enzyme activities including SOD, CAT, and POD. The ROS and cell death were analyzed by flow cytometry with double staining of propidium iodide (PI) and 2’,7’-dichlorofluorescein diacetate (DCFH-DA) according to the previous study [39]. For other tests, the yeast cells were crushed by ultrasound at 500 W and 4 °C for 30 min (10 s/10 s) in PBS. After the samples were centrifuged for 20 min at 12,000 r/min and 4 °C, the supernatant was used to measure MDA, protein carbonyls, and antioxidant enzyme activities as described previously [18].

### Statistical analysis

The transcriptome experiment were performed with two parallel. DEGs were selected by DESeq2 (v1.24.0) with the threshold of |log_2_FC|> 1 and *P*-adjust < 0.05, which were then performed by the GO, COG, and KEGG pathway analysis, respectively. The metabolome experiment were performed with three parallel. The metabolites with the threshold of VIP > 1, *P* value < 0.05, and |log_2_FC|> 1 were considered as DMs, which were further annotated using the HMDB database and KEGG pathway database. The other experiments were performed in triplicate and the data were expressed as mean ± standard deviation. The differences among data were determined by one-way analysis of variance with multiple comparison Tukey tests. PCA was used to evaluate the similarity of metabolome in different groups. The heatmaps of the DEGs and DMs were constructed using Heml (v1.0.3.7). The metabolic network map of *P. kudriavzevii* was constructed based on the KEGG pathways. The key enzymes and metabolites represented in the metabolic network map were, respectively, from the heatmaps of the DEGs and DMs.

## Supplementary Information

**Additional file 1: Table S1.** Summary of RNA-Seq data obtained in this study. **Figure S1.** Effect of salt stress on global transcriptome changes of *P. kudriavzevii* under heat stress. (A) Volcano plots of DEGs in T45 vs T45S100 and T45 vs T45S300 comparison groups. Red diamonds and green squares, respectively, denote the up-regulated and down-regulated DEGs (|log_2_FC|> 1 and P-adjust < 0.05) in the T45S100 and T45S300 groups, compared with those in the T45 group. Clustered (B) GO terms, (C) COG categories, and (D) KEGG categories of DEGs in T45 vs T45S100 and T45 vs T45S300 comparison groups. **Figure S2.** Effect of salt stress on global metabolome changes of *P. kudriavzevii* under heat stress. (A) PCA score plots of metabolome in different groups. (B) Volcano plots of DMs in T45 vs T45S100 and T45 vs T45S300 comparison groups. Red and green dots, respectively, denote the up-regulated and down-regulated DMs (VIP > 1, *P* value < 0.05, and |log_2_FC|> 1) in the T45S100 and T45S300 groups, compared with those in the T45 group. (C) Venn diagram of DMs in T45 vs T45S100 and T45 vs T45S300 comparison groups. Clustered (D) HMDB categories and (E) KEGG pathways of DMs in T45 vs T45S100 and T45 vs T45S300 comparison groups. **Table S2.** Key genes related to the improvement of thermotolerance and high-temperature bioethanol production of *P. kudriavzevii* by salt stress. **Table S3.** Key metabolites related to the improvement of thermotolerance and high-temperature bioethanol production of *P. kudriavzevii* by salt stress.

## Data Availability

All data generated or analyzed during this study are included in this published article and its additional information files.

## Abbreviations

NaCl: Sodium chloride
ATP: Adenosine triphosphate
HSPs: Heat shock proteins
ROS: Reactive oxygen species
GSH: Glutathione
GSSG: Glutathione disulfide
GST: Glutathione S-transferase
SEM: Scanning electron microscope
RNA-Seq: Ribose nucleic acid sequencing
DEGs: Differentially expressed genes
GO: Gene ontology
COG: Cluster of orthologousgroups
KEGG: Kyoto Encyclopedia of Genes and Genomes
TCA: Tricarboxylic acid
GMP: Guanosine monophosphate
CTP: Cytidine triphosphate
PCA: Principal component analysis
DMs: Differential metabolites
HMDB: Human Metabolome Database
NAD: Nicotinamide adenine dinucleotide
NADH: Reduced form of nicotinamide adenine dinucleotide
NADP: Nicotinamide adenine dinucleotide phosphate
CoA: Coenzyme A
MDA: Malonaldehyde
SOD: Superoxide dismutase
POD: Peroxidase
CAT: Catalase
UFAs: Unsaturated fatty acids
PUFAs: Polyunsaturated fatty acids
16(R)-HETE: 16-Hydroxy-5,8,11,14- eicosatetraenoic acid
5-oxoETE: 5-Ketoeicosatetraenoic acid
9-oxo-ODE: (10E,12Z)-9-Oxooctadeca-10,12-dienoic acid
H_2_O_2_: Hydrogen peroxide
YEPD: Yeast extract–peptone–dextrose
VIP: Variable importance in the projection
FC: Fold change
PI: Propidium iodide
DCFH-DA: 2’,7’-Dichlorofluorescein diacetate
DCF: 2’,7’-Dichlorofluorescein
