# Supplementary material for: Salt stress improves thermotolerance and high-temperature bioethanol production of multi-stress-tolerant Pichia kudriavzevii by stimulating intracellular metabolism and inhibiting oxidative damage
Source: Biotechnol Biofuels. 2021 Nov 25;14:222. doi: 10.1186/s13068-021-02071-0 (PMC8613974; doi:10.1186/s13068-021-02071-0)
Supplement: Supplementary file 1 — Additional file 1: Table S1. Summary of RNA-Seq data obtained in this study. Figure S1. Effect of salt stress on global transcriptome changes of P. kudriavzevii under heat stress. (A) Volcano plots of DEGs in T45 vs T45S100 and T45 vs T45S300 comparison groups. Red diamonds and green squares, respectively, denote the up-regulated and down-regulated DEGs (|log2FC|> 1 and P-adjust < 0.05) in the T45S100 and T45S300 groups, compared with those in the T45 group. Clustered (B) GO terms, (C) COG categories, and (D) KEGG categories of DEGs in T45 vs T45S100 and T45 vs T45S300 comparison groups. Figure S2. Effect of salt stress on global metabolome changes of P. kudriavzevii under heat stress. (A) PCA score plots of metabolome in different groups. (B) Volcano plots of DMs in T45 vs T45S100 and T45 vs T45S300 comparison groups. Red and green dots, respectively, denote the up-regulated and down-regulated DMs (VIP > 1, P value < 0.05, and |log2FC|> 1) in the T45S100 and T45S300 groups, compared with those in the T45 group. (C) Venn diagram of DMs in T45 vs T45S100 and T45 vs T45S300 comparison groups. Clustered (D) HMDB categories and (E) KEGG pathways of DMs in T45 vs T45S100 and T45 vs T45S300 comparison groups. Table S2. Key genes related to the improvement of thermotolerance and high-temperature bioethanol production of P. kudriavzevii by salt stress. Table S3. Key metabolites related to the improvement of thermotolerance and high-temperature bioethanol production of P. kudriavzevii by salt stress. [file 13068_2021_2071_MOESM1_ESM.doc]

**Additional Data**

**Salt stress improves** **thermotolerance and high-temperature bioethanol production of** **multi-stress-tolerant *Pichia kudriavzevii* by stimulating** **intracellular metabolism and inhibiting** **oxidative damage**

Chunsheng Li^1,2,3^, Qiuying Liu^1^, Yueqi Wang^1,2,3^, Xianqing Yang^1^*, Shengjun Chen^1^, Yongqiang Zhao^1,2,3^, Yanyan Wu^1^, Laihao Li^1^

^1^Key Laboratory of Aquatic Product Processing, Ministry of Agriculture and Rural Affairs, National R&D Center for Aquatic Product Processing, South China Sea Fisheries Research Institute, Chinese Academy of Fishery Sciences, Guangzhou 510300, China

^2^Co-Innovation Center of Jiangsu Marine Bio-industry Technology, Jiangsu Ocean University, Lianyungang 222005, PR China

^3^Collaborative Innovation Center of Seafood Deep Processing, Dalian Polytechnic University, Dalian 116034, PR China

* Corresponding author:

Xianqing Yang (E-mail: yangxq@scsfri.ac.cn)

Address: Key Laboratory of Aquatic Product Processing, Ministry of Agriculture and Rural Affairs, National R&D Center for Aquatic Product Processing, South China Sea Fisheries Research Institute, Chinese Academy of Fishery Sciences, Guangzhou 510300, China.

Fax: +86 20-82031851.

Telephone: +86 20-89108310.

**Table S1** Summary of RNA-Seq data obtained in this study.

|  | T45a | T45b | T45S100a | T45S100b | T45S300a | T45S300b |
| --- | --- | --- | --- | --- | --- | --- |
| Total raw reads (Mb) | 46.07 | 43.51 | 44.88 | 49.35 | 57.21 | 48.87 |
| Total raw bases (Gb) | 6.96 | 6.57 | 6.78 | 7.45 | 8.63 | 7.38 |
| Total clean reads (Mb) | 45.54 | 42.94 | 44.31 | 48.69 | 56.55 | 48.32 |
| Total clean bases (Gb) | 6.77 | 6.41 | 6.60 | 7.23 | 8.38 | 7.15 |
| Clean reads Q20 (%) | 98.07 | 98.50 | 98.54 | 98.58 | 98.49 | 98.69 |
| Clean reads Q30 (%) | 93.94 | 95.18 | 95.29 | 95.36 | 95.13 | 95.66 |
| GC content (%) | 44.86 | 45.16 | 45.18 | 45.53 | 46.74 | 47.25 |
| Total mapped reads (%) | 96.28 | 96.70 | 96.55 | 96.48 | 96.55 | 96.84 |
| Multiple mapped reads (%) | 31.06 | 33.40 | 32.59 | 34.29 | 43.45 | 47.28 |
| Unique mapped reads (%) | 65.22 | 63.30 | 63.97 | 62.19 | 53.09 | 49.56 |


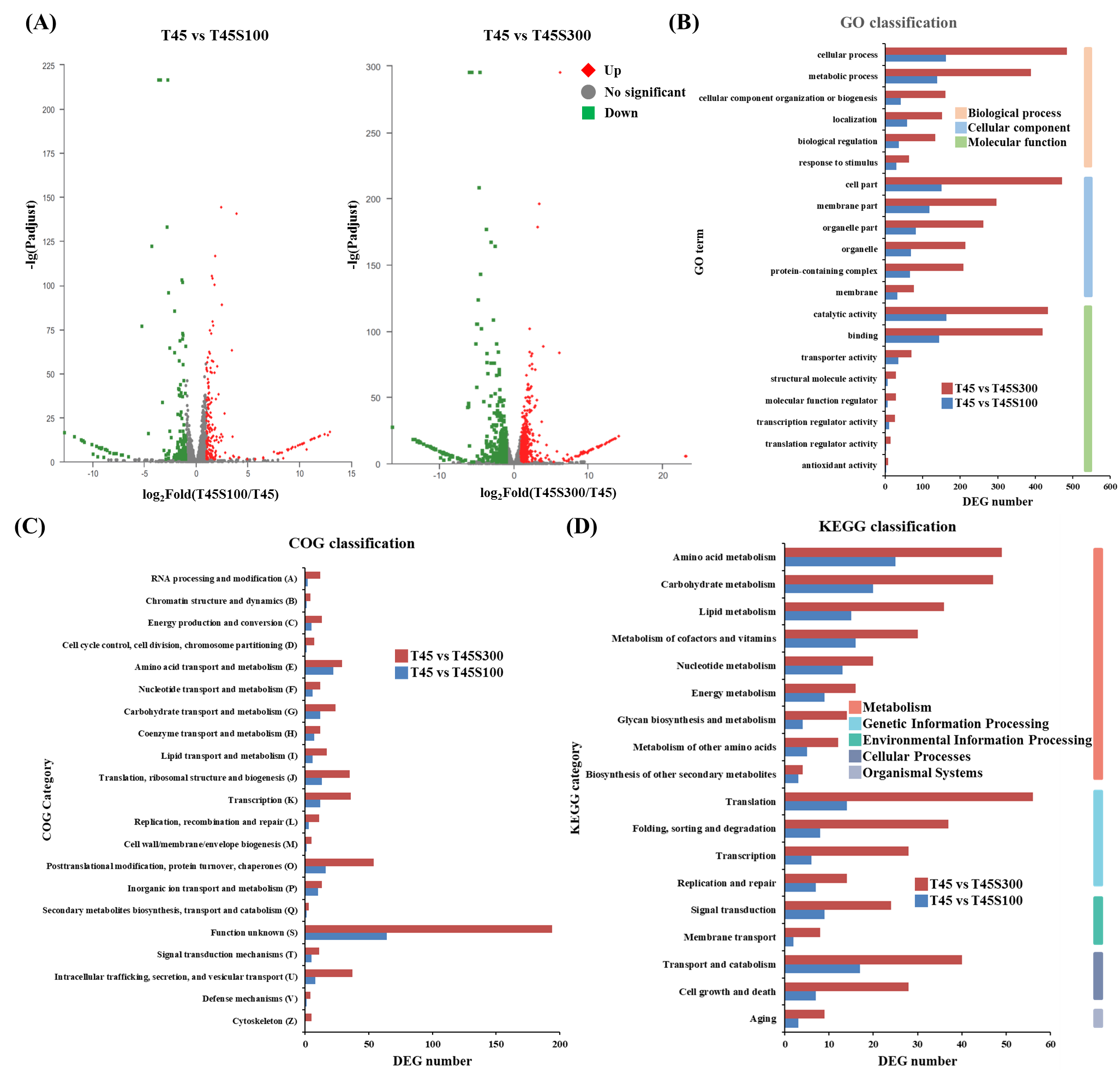


**Figure S1.** Effect of salt stress on global transcriptome changes of *P. kudriavzevii* under heat stress. (A) Volcano plots of DEGs in T45 vs T45S100 and T45 vs T45S300 comparison groups. Red diamonds and green squares respectively denote the up-regulated and down-regulated DEGs (|log_2_FC|>1 and P-adjust<0.05) in the T45S100 and T45S300 groups, compared with those in the T45 group. Clustered (B) GO terms, (C) COG categories, and (D) KEGG categories of DEGs in T45 vs T45S100 and T45 vs T45S300 comparison groups.


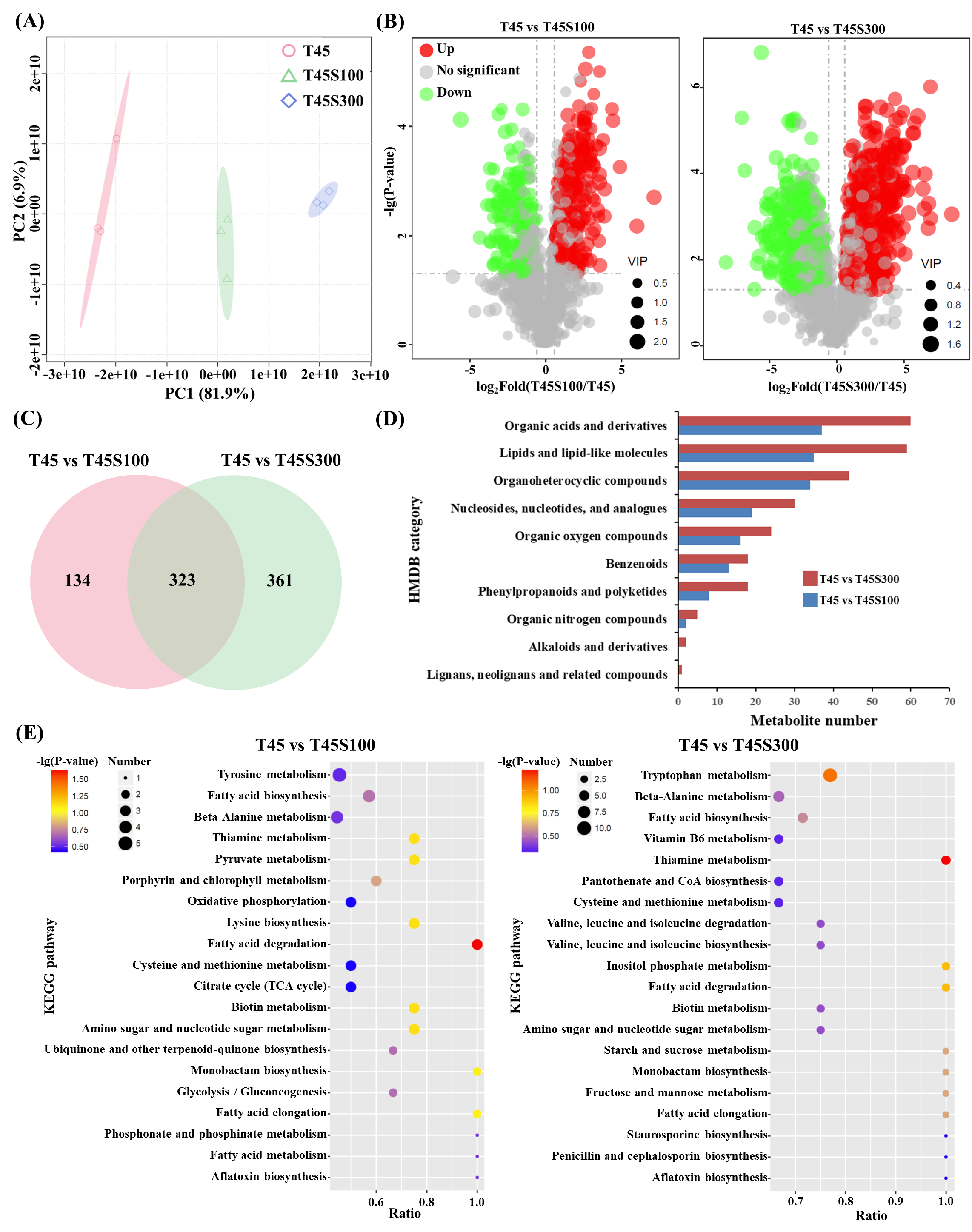


**Figure S2.** Effect of salt stress on global metabolome changes of *P. kudriavzevii* under heat stress. (A) PCA score plots of metabolome in different groups. (B) Volcano plots of DMs in T45 vs T45S100 and T45 vs T45S300 comparison groups. Red and green dots respectively denote the up-regulated and down-regulated DMs (VIP>1, P-value<0.05, and |log_2_FC|>1) in the T45S100 and T45S300 groups, compared with those in the T45 group. (C) Venn diagram of DMs in T45 vs T45S100 and T45 vs T45S300 comparison groups. Clustered (D) HMDB categories and (E) KEGG pathways of DMs in T45 vs T45S100 and T45 vs T45S300 comparison groups.

**Table S2** Key genes related to the improvement of thermotolerance and high-temperature bioethanol production of *P. kudriavzevii* by salt stress.

| Gene symbol | Gene description | log_2_(T45S100/T45) | log_2_(T45S300/T45) |
| --- | --- | --- | --- |
| Carbohydrate metabolism | | | |
| BOH78_1152 | Alcohol dehydrogenase 4, mitochondrial | 0.78 | 5.06 |
| BOH78_4489 | NADP-dependent alcohol dehydrogenase 7 | 1.00 | 1.29 |
| BOH78_5258 | NADP-dependent alcohol dehydrogenase 6 | 2.54 | 1.52 |
| BOH78_4213 | NADP-dependent alcohol dehydrogenase 6 | 1.15 | 0.27 |
| BOH78_0056 | Aldehyde dehydrogenase 5, mitochondrial | 0.00 | -1.35 |
| BOH78_1364 | Aldehyde dehydrogenase 5, mitochondrial | -0.50 | -2.68 |
| BOH78_4243 | Pyruvate decarboxylase isozyme 3 | 0.09 | -1.17 |
| BOH78_1895 | D-lactate dehydrogenase [cytochrome], mitochondrial | 0.00 | 9.62 |
| BOH78_0689 | D-lactate dehydrogenase [cytochrome], mitochondrial | 1.15 | 0.71 |
| BOH78_4711 | D-lactate dehydrogenase [cytochrome], mitochondrial | 1.09 | 0.71 |
| BOH78_0831 | Phosphoenolpyruvate carboxykinase [ATP] | 0.63 | 2.35 |
| BOH78_5016 | Pyruvate kinase | 1.02 | 0.62 |
| BOH78_3854 | Malate dehydrogenase, mitochondrial | 0.04 | 1.41 |
| BOH78_3867 | Succinate dehydrogenase [ubiquinone] cytochrome b small subunit | 0.71 | 1.11 |
| BOH78_1753 | Ribose-5-phosphate isomerase | 1.07 | 1.37 |
| BOH78_0868 | Ribose-phosphate pyrophosphokinase 1 | 0.53 | 1.57 |
| BOH78_1358 | Ribose-phosphate pyrophosphokinase 1 | 1.12 | 1.79 |
| BOH78_2383 | Ribose-phosphate pyrophosphokinase 5 | 0.00 | 12.96 |
| BOH78_1531 | Protoheme IX farnesyltransferase, mitochondrial | 1.53 | 3.24 |
| BOH78_4673 | Cytochrome c oxidase polypeptide 5B, mitochondrial | 0.53 | 1.49 |
| BOH78_1614 | Cytochrome c oxidase subunit 6, mitochondrial | 0.18 | 1.95 |
| BOH78_2664 | Cytochrome c oxidase subunit 4, mitochondrial | 0.51 | 1.07 |
| BOH78_1969 | Cytochrome c oxidase subunit 7 | 0.42 | 1.13 |
| BOH78_3744 | ATP synthase subunit g, mitochondrial | 0.23 | 1.06 |
| Nucleotide metabolism | | | |
| BOH78_2861 | GMP synthase [glutamine-hydrolyzing] | 0.87 | 1.20 |
| BOH78_5363 | Adenylosuccinate lyase | 1.22 | -1.25 |
| BOH78_2114 | Adenylosuccinate synthetase | 1.53 | -0.3 |
| BOH78_3851 | Adenine phosphoribosyltransferase | 0.89 | 1.5 |
| BOH78_4903 | Adenine deaminase | 1.48 | 0.54 |
| BOH78_0903 | Inosine-5'-monophosphate dehydrogenase | 0.89 | 1.94 |
| BOH78_1980 | Carbamoyl-phosphate synthase arginine-specific small chain | 0.78 | 1.94 |
| BOH78_0312 | Carbamoyl-phosphate synthase arginine-specific large chain | 0.18 | 1.47 |
| BOH78_0643 | Dihydroorotase | -0.61 | 2.14 |
| BOH78_2819 | Orotate phosphoribosyltransferase | 0.93 | 1.22 |
| BOH78_2964 | Uridine kinase | 0.93 | 1.51 |
| BOH78_3335 | Deoxycytidylate deaminase | 0.78 | 1.06 |
| Amino acid metabolism | | | |
| BOH78_0416 | Prephenate dehydrogenase [NADP(+)] | 11.84 | 12.46 |
| BOH78_0269 | Aromatic/aminoadipate aminotransferase 1 | 0.90 | 1.07 |
| BOH78_4469 | D-3-phosphoglycerate dehydrogenase 1 | 1.10 | 0.29 |
| BOH78_2637 | Phosphoserine phosphatase | 1.20 | 1.19 |
| BOH78_1509 | Serine hydroxymethyltransferase, cytosolic | 1.62 | 0.47 |
| BOH78_3435 | Low-specificity L-threonine aldolase | 1.06 | 1.44 |
| BOH78_3264 | Cysteine synthase | 0.41 | 1.45 |
| BOH78_3208 | putative cysteine synthase | 3.66 | 4.41 |
| BOH78_3644 | Phosphoribosyl isomerase | 1.05 | 1.73 |
| BOH78_4173 | Imidazoleglycerol-phosphate dehydratase | 0.33 | 1.63 |
| BOH78_4226 | Argininosuccinate synthase | 0.94 | 2.47 |
| BOH78_2917 | Argininosuccinate lyase | 0.56 | 1.46 |
| BOH78_1802 | Homoisocitrate dehydrogenase, mitochondrial | 1.28 | 1.01 |
| BOH78_1841 | Protein ARG5,6, mitochondrial | 0.82 | 2.41 |
| BOH78_4226 | Argininosuccinate synthase | 0.94 | 2.47 |
| BOH78_4829 | Arginine biosynthesis bifunctional protein ArgJ, mitochondrial | 1.21 | 2.01 |
| BOH78_0346 | Ornithine carbamoyltransferase | 0.64 | 2.09 |
| BOH78_4493 | Arginase | 0.05 | -1.92 |
| BOH78_2917 | Argininosuccinate lyase | 0.56 | 1.46 |
| BOH78_2352 | Guanidinobutyrase | 1.41 | 2.30 |
| BOH78_5037 | Guanidinobutyrase | 10.07 | 22.99 |
| BOH78_1556 | Spermidine synthase | 1.80 | 1.05 |
| BOH78_3753 | Spermine synthase | 0.54 | 1.19 |
| Lipid metabolism | | | |
| BOH78_1417 | Fatty acid synthase subunit alpha | 0.37 | 1.72 |
| BOH78_2482 | Fatty acid synthase subunit beta | 0.44 | 1.19 |
| BOH78_2959 | 3-oxoacyl-[acyl-carrier-protein] synthase, mitochondrial | 0.60 | 1.54 |
| BOH78_3426 | Glycerol 2-dehydrogenase (NADP(+)) | 1.24 | -1.77 |
| BOH78_0965 | Dihydroxyacetone kinase | -0.56 | -1.35 |
| BOH78_3328 | Glycerol-3-phosphate O-acyltransferase 1 | 0.50 | 1.09 |
| BOH78_0694 | Protein PNS1 | 0.65 | 1.85 |
| BOH78_2565 | Ubiquitin-like protein SMT3 | -0.11 | -1.07 |
| BOH78_3324 | CTP-dependent diacylglycerol kinase 1 | 0.29 | 1.05 |
| BOH78_3215 | Phosphatidate cytidylyltransferase | 1.80 | 2.53 |
| BOH78_0287 | Cholinephosphotransferase 1 | 9.83 | 0.00 |
| BOH78_3170 | 3-ketodihydrosphingosine reductase | 0.63 | 1.10 |
| BOH78_4629 | Sphingolipid C4-hydroxylase SUR2 | 1.03 | 1.04 |
| BOH78_5460 | Dihydroceramide delta(4)-desaturase | 0.43 | 1.76 |
| BOH78_4944 | Ceramide glucosyltransferase | 1.31 | 1.25 |
| BOH78_2431 | Methylsterol monooxygenase | 0.55 | 1.31 |
| BOH78_4121 | Acyl-CoA desaturase 1 | 0.75 | 1.98 |
| BOH78_1897 | Acyl-CoA desaturase | -7.97 | 1.19 |
| BOH78_1107 | Delta(12) fatty acid desaturase | 0.45 | 1.49 |
| BOH78_1106 | Delta(12) fatty acid desaturase | 0.62 | 3.06 |
| Transcription | | | |
| BOH78_4804 | DNA-directed RNA polymerase I subunit RPA2 | 1.22 | 2.22 |
| BOH78_3645 | DNA-directed RNA polymerase II subunit RPB3 | 0.12 | 1.41 |
| BOH78_2785 | DNA-directed RNA polymerases I and III subunit RPAC1 | 0.69 | 1.29 |
| BOH78_0356 | DNA-directed RNA polymerase III subunit RPC1 | 0.25 | 1.02 |
| BOH78_2225 | DNA-directed RNA polymerase I subunit RPA43 | 8.97 | 11.83 |
| BOH78_0953 | DNA-directed RNA polymerases I, II, and III subunit RPABC5 | 0.68 | 1.27 |
| BOH78_3149 | DNA-directed RNA polymerase I subunit RPA34 | 0.66 | 1.39 |
| BOH78_2728 | DNA-directed RNA polymerase I subunit RPA34 | 0.66 | 1.39 |
| BOH78_3487 | DNA-directed RNA polymerases I, II, and III subunit RPABC1 | 0.25 | 1.44 |
| BOH78_4398 | DNA-directed RNA polymerase III subunit RPC5 | 1.39 | 2.22 |
| BOH78_4772 | DNA-directed RNA polymerase III subunit RPC8 | 4.54 | 7.69 |
| BOH78_5290 | DNA-directed RNA polymerase I subunit RPA34 | 0.49 | 1.14 |
| BOH78_3164 | DNA-directed RNA polymerase II subunit RPB9 | 0.54 | 3.55 |
| BOH78_3107 | DNA-directed RNA polymerase I subunit RPA12 | 0.43 | 2.02 |
| BOH78_3459 | 13 kDa ribonucleoprotein-associated protein | 0.83 | 2.11 |
| BOH78_0354 | Pre-mRNA-processing factor 17 | 0.94 | 1.37 |
| BOH78_3131 | Pre-mRNA-splicing factor ISY1 | 0.62 | 1.36 |
| BOH78_0971 | Pre-mRNA-splicing ATP-dependent RNA helicase PRP28 | 1.37 | 2.44 |
| BOH78_1582 | U2 small nuclear ribonucleoprotein A' | 0.45 | 1.89 |
| BOH78_0497 | Small nuclear ribonucleoprotein Sm D1 | 5.74 | 7.72 |
| BOH78_0050 | Spliceosomal protein DIB1 | -0.73 | 1.33 |
| BOH78_1470 | Pre-mRNA-splicing factor RDS3 | 0.48 | 1.04 |
| BOH78_3759 | Pre-mRNA-splicing factor CWC15 | 0.28 | 1.31 |
| Translation | | | |
| BOH78_3446 | Ribosomal RNA-processing protein 7 | 0.10 | 1.36 |
| BOH78_0406 | rRNA-processing protein FCF1 | 8.47 | 11.63 |
| BOH78_3398 | Nucleolar GTP-binding protein 1 | 1.46 | 2.13 |
| BOH78_1859 | Eukaryotic translation initiation factor 6 | 0.77 | 1.87 |
| BOH78_2708 | rRNA 2'-O-methyltransferase fibrillarin | 0.66 | 1.53 |
| BOH78_4275 | Nucleolar protein 56 | 0.85 | 1.42 |
| BOH78_4964 | H/ACA ribonucleoprotein complex subunit 1 | 0.41 | 1.06 |
| BOH78_4856 | GTP-binding nuclear protein GSP1/Ran | 0.39 | 1.18 |
| BOH78_1577 | Casein kinase I hhp1 | 0.47 | 1.76 |
| BOH78_0298 | Casein kinase II subunit beta | -0.01 | 1.39 |
| BOH78_1151 | Serine/threonine-protein kinase RIO2 | 0.49 | 1.24 |
| BOH78_4503 | H/ACA ribonucleoprotein complex subunit 2 | 0.20 | 1.08 |
| BOH78_4176 | Retrograde regulation protein 1 | 1.68 | 2.21 |
| BOH78_2787 | Nucleolar protein 6 | 0.14 | 1.00 |
| BOH78_4923 | Ribosome biogenesis protein BMS1 | 0.00 | 1.33 |
| BOH78_3532 | RNA 3'-terminal phosphate cyclase-like protein | 0.87 | 1.15 |
| BOH78_4651 | Ribonuclease P protein subunit p29 | 0.65 | 1.27 |
| BOH78_4116 | Nucleolar protein 58 | 0.08 | 1.59 |
| BOH78_5324 | Large subunit GTPase 1 | -7.08 | -6.96 |
| BOH78_2572 | N-glycosylation protein EOS1 | 1.33 | 2.10 |
| BOH78_0633 | Ribosome biogenesis protein RLP24 | 0.76 | 1.22 |
| BOH78_2083 | Tyrosine--tRNA ligase, cytoplasmic | 0.35 | 1.07 |
| BOH78_2773 | putative lysine--tRNA ligase, cytoplasmic | -0.20 | 1.09 |
| BOH78_3193 | Aspartate--tRNA ligase, mitochondrial | -0.05 | 1.13 |
| BOH78_1411 | Arginine--tRNA ligase, cytoplasmic | 1.67 | 1.47 |
| BOH78_0426 | putative proline--tRNA ligase, mitochondrial | -0.98 | -1.38 |
| BOH78_2176 | Methionyl-tRNA formyltransferase | 0.49 | 1.18 |
| BOH78_0077 | Serine--tRNA ligase, cytoplasmic | -1.52 | -3.96 |
| Replication and repair | | | |
| BOH78_2483 | DNA ligase 1 | -9.15 | -9.04 |
| BOH78_1122 | Proliferating cell nuclear antigen | -0.71 | -1.10 |
| BOH78_0491 | DNA polymerase epsilon subunit B | -0.14 | -1.08 |
| BOH78_0551 | DNA mismatch repair protein MSH6 | -1.02 | -2.01 |
| BOH78_4451 | DNA mismatch repair protein MLH1 | -0.92 | -1.11 |
| BOH78_1425 | DNA mismatch repair protein MSH3 | -1.13 | -2.09 |
| BOH78_1846 | DNA mismatch repair protein PMS1 | -1.17 | -1.17 |
| BOH78_1028 | Replication factor C subunit 5 | -1.51 | -1.37 |
| BOH78_0491 | DNA polymerase epsilon subunit B | -0.14 | -1.08 |
| BOH78_0622 | RNA polymerase II transcription factor B subunit 1 | -1.15 | -1.45 |
| BOH78_2370 | DNA repair protein RAD1 | -1.08 | -1.09 |
| BOH78_1625 | DNA repair and recombination protein RDH54 | -0.81 | -1.05 |
| BOH78_1060 | Crossover junction endonuclease EME1 | -0.85 | -1.87 |
| BOH78_0180 | Serine/threonine-protein kinase TEL1 | -0.88 | -1.05 |
| Aging | | | |
| BOH78_2676 | Heat shock protein SSA3 | 0.56 | 3.17 |
| BOH78_3584 | Heat shock protein SSA2 | -0.42 | 1.46 |
| BOH78_3967 | Heat shock protein 78, mitochondrial | -0.30 | 1.15 |
| BOH78_1681 | Superoxide dismutase [Mn], mitochondrial | 1.12 | 1.39 |
| BOH78_3319 | Superoxide dismutase [Fe] | 0.24 | 1.05 |

**Table S3** Key metabolites related to the improvement of thermotolerance and high-temperature bioethanol production of *P. kudriavzevii* by salt stress.

| Metabolite name | log_2_(T45S100/T45) | log_2_(T45S300/T45) |
| --- | --- | --- |
| Carbohydrate metabolism | | |
| D-Glucose 6-phosphate | -0.73 | 1.66 |
| D-Glyceraldehyde 3-phosphate | -0.26 | 2.60 |
| 3-Phosphoglyceric acid | -1.61 | 0.10 |
| Acetyl-CoA | 1.84 | 2.72 |
| NAD | 1.15 | 2.19 |
| NADH | 2.84 | 4.55 |
| CoA-SH | 2.33 | 2.21 |
| 2-Oxoglutarate | 1.62 | 1.72 |
| Succinate | 2.03 | 2.07 |
| NADP | 0.55 | 1.29 |
| FAD | 0.41 | 1.41 |
| ATP | 1.21 | 2.60 |
| ADP | 1.69 | 2.70 |
| α,α-Trehalose | 0.97 | 3.85 |
| Nucleotide metabolism | | |
| L-Dihydroorotate | 0.96 | 6.81 |
| Orotate | 1.81 | 8.54 |
| AMP | 0.34 | 0.99 |
| Adenosine | -0.25 | -1.09 |
| Adenine | 1.62 | 1.49 |
| Inosine | 2.45 | 0.71 |
| IMP | 2.17 | 0.86 |
| GDP | 0.57 | 0.62 |
| cAMP | 1.12 | 2.57 |
| cGMP | 0.95 | 0.98 |
| Cytidine | 0.86 | 2.50 |
| CMP | -0.01 | 0.62 |
| CDP | 1.16 | 2.81 |
| UTP | 0.00 | 2.21 |
| Thymine | 1.00 | 1.87 |
| Thymidine | 0.48 | 1.14 |
| dTMP | 1.01 | 1.73 |
| TMP | 2.53 | 3.88 |
| 2'-Deoxyuridine | 0.59 | 1.82 |
| 2'-Deoxycytidine | 1.35 | 6.55 |
| Amino acid metabolism | | |
| GSSG | 1.47 | 2.41 |
| GSH | 1.67 | 2.53 |
| gamma-Glutamylcysteine | 1.62 | 1.15 |
| S-Lactoylglutathione | 4.37 | 6.40 |
| N-Acetyl-L-glutamine | 0.86 | 3.24 |
| N2-Acetyl-L-ornithine | 0.96 | 2.31 |
| L-Argininosuccinate | 0.68 | 5.08 |
| Ornithine | 0.65 | 1.63 |
| Spermidine | 1.18 | 2.47 |
| Spermine | 3.86 | 3.45 |
| Phenylalanine | 0.68 | 0.97 |
| L-Tyrosine | 2.37 | 2.93 |
| L-Cysteine | 0.20 | 0.63 |
| Threonine | 0.03 | 1.57 |
| O-Phospho-L-serine | 1.11 | 2.58 |
| Tryptophan | 0.76 | 1.11 |
| L-Histidine | 0.24 | 1.15 |
| L-Glutamte | -0.09 | 1.33 |
| Aspartate | -0.64 | 2.18 |
| L-Valine | 0.64 | 1.15 |
| Lipid metabolism | | |
| Octanoic acid | -0.39 | 2.82 |
| Decanoic acid | -0.78 | 2.48 |
| Heptadecanoic Acid | 2.14 | 1.67 |
| Stearic acid | -0.42 | -2.43 |
| Sphinganine | 1.48 | 0.84 |
| N-Acetylsphingosine | 1.33 | 2.02 |
| Sphingosine | -2.56 | -3.00 |
| Psychosine | -6.12 | -6.11 |
| Glycerophosphorylcholine | 0.31 | 1.14 |
| 16(R)-HETE | 1.20 | 1.39 |
| 8Z,11Z,14Z-Eicosatrienoic acid | 1.45 | 2.28 |
| 5-OxoETE | 2.93 | 2.37 |
| 9-Hpode | 1.01 | 0.70 |
| 9-Oxo-ODE | 1.60 | 2.24 |
| cis-2-Decenoic acid | 0.14 | 3.07 |
| 9,10-Dihome | 1.41 | 1.41 |
